# Characterization of the Olfactory Receptors Expressed in Human Spermatozoa

**DOI:** 10.3389/fmolb.2015.00073

**Published:** 2016-01-07

**Authors:** Caroline Flegel, Felix Vogel, Adrian Hofreuter, Benjamin S. P. Schreiner, Sandra Osthold, Sophie Veitinger, Christian Becker, Norbert H. Brockmeyer, Michael Muschol, Gunther Wennemuth, Janine Altmüller, Hanns Hatt, Günter Gisselmann

**Affiliations:** ^1^Department of Cell Physiology, Ruhr-University BochumBochum, Germany; ^2^Cologne Center for Genomics, University of KölnKöln, Germany; ^3^Department of Dermatology and Venereology, Center for Sexual Health and Medicine, Ruhr University BochumBochum, Germany; ^4^Competence Network for HIV/AIDS, Ruhr University BochumBochum, Germany; ^5^Institute of Anatomy, University Hospital EssenEssen, Germany

**Keywords:** spermatozoa, odorant receptor, deep sequencing, antisense transcripts, signal transduction

## Abstract

The detection of external cues is fundamental for human spermatozoa to locate the oocyte in the female reproductive tract. This task requires a specific chemoreceptor repertoire that is expressed on the surface of human spermatozoa, which is not fully identified to date. Olfactory receptors (ORs) are candidate molecules and have been attributed to be involved in sperm chemotaxis and chemokinesis, indicating an important role in mammalian spermatozoa. An increasing importance has been suggested for spermatozoal RNA, which led us to investigate the expression of all 387 OR genes. This study provides the first comprehensive analysis of OR transcripts in human spermatozoa of several individuals by RNA-Seq. We detected 91 different transcripts in the spermatozoa samples that could be aligned to annotated OR genes. Using stranded mRNA-Seq, we detected a class of these putative OR transcripts in an antisense orientation, indicating a different function, rather than coding for a functional OR protein. Nevertheless, we were able to detect OR proteins in various compartments of human spermatozoa, indicating distinct functions in human sperm. A panel of various OR ligands induced Ca^2+^ signals in human spermatozoa, which could be inhibited by mibefradil. This study indicates that a variety of ORs are expressed at the mRNA and protein level in human spermatozoa.

## Introduction

A new and unique individual can be conceived when a spermatozoon has transferred its genetic material into an oocyte. Prior to fertilization of the oocyte, the spermatozoa in the female reproductive tract become hyperactivated and undergo capacitation and an acrosome reaction. On their long path to the oocyte, the spermatozoa are exposed to a number of different chemical stimuli originating from their environment, the oocyte, or its surrounding cells (Sun et al., 2005). To date, a variety of different sperm attractants of aquatic and terrestrial organisms were identified (Riffell et al., 2002) including odorants (Spehr et al., 2003). Additionally, further unidentified chemosignals in the different fluids of the female reproductive tract may participate in processes that prime sperm for fertilization. Chemosignals can influence sperm motility (chemokinesis) and induce chemo- and thermotaxis and thus optimize the interaction between the female and male germ cells. However, the effects of such environmental signals have been rudimentarily explored. Different studies have already described receptors that are responsible for the detection of chemical molecules in mammalian spermatozoa (Spehr et al., 2003; Eisenbach and Giojalas, 2006; Martínez-López et al., 2011; Veitinger et al., 2011; Brenker et al., 2012; Meyer et al., 2012; Schiffer et al., 2014). However, the detection of chemical cues by human spermatozoa has not been clarified in detail.

We and others have previously suggested that chemoreceptors are involved in the processing of chemical molecules in spermatozoa (Spehr et al., 2003; Fukuda and Touhara, 2006; Veitinger et al., 2011; Meyer et al., 2012). Olfactory receptors (ORs) form the largest group of human chemoreceptors. The receptors consist of ~400 functional genes in the human genome (Firestein, 2001; Glusman et al., 2001). Parmentier and coworkers reported that ORs are also expressed at RNA level in the mammalian testis (Parmentier et al., 1992). Until today, many studies have reported the existence of OR transcripts in the mammalian testis, pre- and postmeiotic germ cells, and mature spermatozoa (Parmentier et al., 1992; Walensky et al., 1995; Vanderhaeghen et al., 1997a,b; Zhang and Firestein, 2002; Spehr et al., 2003; Fukuda et al., 2004; Feldmesser et al., 2006; Fukuda and Touhara, 2006; Zhang et al., 2007; Flegel et al., 2013). In a comprehensive RNA-Seq study, we clearly identified the human testis as the tissue with the highest number of ectopically expressed OR transcripts (55 different OR transcripts) in comparison to the other 15 human tissues investigated (Flegel et al., 2013). Moreover, we showed that OR1D2 (OR17-4) can be activated by the synthetic odorant bourgeonal (Spehr et al., 2003) and is localized in the midpiece of human spermatozoa (Neuhaus et al., 2006). In addition, OR4D1 and OR7A5 are expressed in human testis, and sperm can be activated by the synthetic as well as the naturally occurring ligands of these two receptors (Veitinger et al., 2011; Hartmann et al., 2013).

An increasing importance has been suggested for spermatozoal RNA (Ostermeier et al., 2004, 2005; Sendler et al., 2013), which prompted us to investigate the expression of OR transcripts in human spermatozoa. RNA present in mature sperm may encode proteins that are involved in past, current, and future processes (Sendler et al., 2013). Interestingly, the presence of OR transcripts has never been shown for human spermatozoa, but only for the testis. This study provides the first comprehensive analysis of chemoreceptor transcripts in human spermatozoa from several individuals by RNA-Seq analysis. Furthermore, we observed a distinct expression pattern for each OR protein and investigated effects of the respective OR ligands.

## Materials and methods

### Human sperm preparation

Human sperm were freshly obtained from young healthy donors who gave informed signed consent. Samples were used anonymously. Sperm collection and analysis was approved by the local ethics committee of the Ruhr-University Bochum (Reg.-Nr. 2231). For RNA isolation, calcium imaging, acrosome assays, and immunocytochemistry experiments motile spermatozoa were obtained as follows. The sperm were handled and prepared as described in Spehr et al. (2003) and Veitinger et al. (2011). Briefly, liquefied semen was overlaid on a two-layer Percoll (cell culture tested, Sigma-Aldrich, MO, USA) density gradient and centrifuged at room temperature for 40 min at 275 g. The pellet was collected, washed in Ringer's solution (140 mM NaCl, 5 mM KCl, 2 mM CaCl_2_, 2 mM MgCl_2_, 10 mM Hepes, 10 mM glucose, pH 7.4), and again centrifuged for 15 min. Then, the motile spermatozoa pellet was resuspended in Ringer's solution and was used for further experiments.

### RNA isolation

The RNA was isolated using the RNeasy Mini Kit (Qiagen, Hilden, Germany) according to the manufacturer's protocol.

### RNA-seq by next generation sequencing

We used the TruSeq™ RNA Sample Prep Kit v2 protocol for standard mRNAseq and the TruSeq™ Stranded mRNA Library Prep Kit (both Illumina, San Diego, USA) for strand specific mRNAseq. RNA-Seq was performed on the Illumina GAIIx (1 × 75 bp reads) and HiSeq 2000 (2 × 101 bp reads) sequencing platforms. For comparison to the spermatozoa transcript expression, we reanalyzed previously published raw data in the same manner as the RNA-Seq data for the spermatozoa samples. We reanalyzed the data from eight different testis samples obtained from the Array Express Archive (http://www.ebi.ac.uk/arrayexpress/; accession number: E-MTAB-1733). The five reference tissues and one testis sample (Testis 1) from the Body Map 2.0 project were obtained from the NCBI GEO database (http://www.ncbi.nlm.nih.gov/gds/; accession number: GSE30611). The data set for Testis sample 2 was obtained from http://www.ncbi.nlm.nih.gov/gds/; accession number: GSE12946 (Wang et al., 2008).

### RNA-seq read alignment using tophat

The RNA-Seq data were analyzed as previously described (Flegel et al., 2013). The reads were aligned to the hg19 reference genome by TopHat v2.0.6. The aligned data were visualized with the Integrative Genomic Viewer (IGV) (Thorvaldsdóttir et al., 2013). The command line used for TopHat was as follows:

tophat - -output-dir <name output> –GTF <hg19refseq.gtf> <indexes> tissue.fq

### Alignment and gene expression assembly using cufflinks

The Cufflinks v1.3.0 software was used to calculate the abundance of the transcripts based on RefSeq gene model as previously described (Flegel et al., 2013). The relative abundance of the transcripts was reported in FPKM (fragments per kilobase of exon per million fragments mapped) units (Trapnell et al., 2010). The Cufflinks parameters are listed below.

cufflinks - -output-dir <name output2> - -GTF hg19refseq.gtf. - -multi-read-correct - -compatible-hits-norm - -minfrags-per-transfrag 1 - -frags-bias-correct <hg19.fa> sorted.bam

### Cell culture and transfection

To test the specificity of OR antibodies, we used Hana3A cells to express recombinant ORs. The Hana3A cells were kindly provided by H. Matsunami (Duke University Medical Center, Durham, NC, USA). Hana3A cells were maintained under standard conditions, as previously described (Wallrabenstein et al., 2013). For antibody tests, the cells were grown on cover slips in 24-well plates and were transfected with Lipofectamine 2000 (Invitrogen, Carlsbad, CA, USA) according to the manufacturer's protocol with 300 ng of the OR plasmid and 60 ng of the mRTP1S plasmid. For the calcium imaging experiments, the Hana3A cells were grown in 35 mm-cell culture dishes (50% confluence) and transfected with the respective OR plasmid (3 μg), and plasmids (pCI) coding for mRTP1S (0.5 μg) and Gα_olf_ (0.5 μg) for 48 h using a standard calcium phosphate precipitation technique (Busse et al., 2014).

For the antibody specificity studies and the deorphanization studies, the respective OR coding sequence was amplified from human genomic DNA using PCR amplification and specific primer pairs (Figure S2). The primer pairs amplified the complete open reading frame and contain EcoRI and NotI or NotI and ApaI restriction sites for further subcloning into the pCI plasmid (Promega, Madison, USA), which contains the coding sequence for the N-terminal rhodopsin tag (rho-tag, first 20 amino acids of rhodopsin). The plasmid constructs and PCR products were verified by Sanger sequencing.

### Immunocytochemical staining

We fixed transfected Hana3A cells grown on cover slips with 4% paraformaldehyde. Purified human sperm were incubated in Ringer's solution and placed on poly-L-lysine-coated (Sigma-Aldrich, MO, USA) cover slips and then fixed with 4% paraformaldehyde. Next, the fixed cells were permeabilized with PBS^−^/^−^+ 0.1% Triton X-100, washed with PBS^−^/^−^, and incubated with blocking reagent (PBS^−^/^−^+ 0.1% Triton X-100, 5% NGS, and 1% fish gelatine) for 1 h. The cells were incubated overnight (4°C) with the primary antibody and then incubated with the indicated Alexa-conjugated secondary antibody (Invitrogen, Carlsbad, USA) and DAPI for 45 min at room temperature in the absence of light. The following antibodies were used: α-OR6B2 (polyclonal, rabbit IgG, 1:100, Novus Biologicals, Cambridge, UK), α-OR10J1 (polyclonal, rabbit IgG, 1:50, Biorbyt, Cambridge, UK), α-OR2H1/2 (polyclonal, rabbit IgG, 1:100, antikoerper-online.de, Aachen, Germany), α-OR3A2 (polyclonal, rabbit IgG, 1:50, Biorbyt, Cambridge, UK), α-OR4N4 (polyclonal, 1:50, rabbit IgG Sigma-Aldrich, MO, USA), α-OR2W3 (polyclonal, 1:50, rabbit IgG Sigma-Aldrich, MO, USA), α-OR51E1 (custom designed, polyclonal, 1:50, rabbit IgG Eurogentec, Seraing, Belgium), α-OR51E2 (custom designed, polyclonal, 1:50, rabbit IgG Eurogentec, Seraing, Belgium), and α-Rhodopsin 4D2, monoclonal, 1:100 (Merck-Millipore, Darmstadt, Germany). The samples were mounted in ProLong Gold (Invitrogen, Carlsbad, USA). The images were obtained using a confocal fluorescent microscope (LSM 510 Meta; Carl Zeiss, Oberkochen, Germany).

### Single cell calcium imaging

The intracellular Ca^2+^ levels in human spermatozoa and Hana3A cells were measured as previously described (Veitinger et al., 2011). The calcium imaging equipment we used consists of an inverted microscope equipped for ratiometric live single cell imaging (Leica, DMI 3000 B) and a monochromator (Polychrome V, TILL Photonics, FEI). The field of view of the cells was randomly selected. The cells were sequentially illuminated at 340 and 380 nm. The substances were applied via a manifold superfusion device that allows an immediate change of solutions. The single sperm calcium imaging experiments were repeated at least three times, using cells from different donors. The recombinant OR calcium imaging measurements were used cells from at least three independent transfections.

### Chemical substances

The odorants used in the study were provided by Sigma-Aldrich, Henkel AG (Düsseldorf, Germany), or Symrise AG (Holzminden, Germany). The stock dilutions were freshly prepared before use with DMSO (maximum DMSO content 0.1%). Other chemical substances were purchased from Sigma-Aldrich (St. Louis, MO, USA).

## Results

### RNA-seq analysis of human spermatozoa and testes

The RNA-Seq data from the human spermatozoa samples were generated using the Illumina sequencing technique. In total, we generated mRNA-Seq data for 10 human spermatozoa samples from four individual donors (Sperm donors 1-4). Four independent semen samples from sperm donors 3 and 4 were sequenced (Sperm donors 3.1-3.4 and 4.1-4-4) (Table 1). Furthermore, we also generated stranded RNA-Seq data from three human spermatozoa samples (Sperm donors 1-S, 4-S, and 5-S) to investigate the spermatozoal transcript structure in more detail (Table 1). The spermatozoa datasets were compared to transcriptomes of 10 different testis samples (Human Bodymap Atlas 2.0, Wang et al., 2008, and Human Protein Atlas) and to a panel of five reference tissues (brain, colon, liver, lung, and skeletal muscle data sets from the Human Bodymap Atlas 2.0). We analyzed the data using TopHat and Cufflinks software as described previously (Flegel et al., 2013). The sequence reads were mapped onto the human reference genome (hg19). The expression intensities of the transcripts were calculated for each sample based on the number of fragments per kilobase of exon per million fragments mapped (FPKM) (Mortazavi et al., 2008). On a rough scale, 1 FPKM corresponds to weak expression, 10 FPKM to moderate expression, and 100 FPKM to a high expression level. The FPKM values for typical housekeeping genes were calculated. For example, the weakly to moderately expressed TATA box binding protein (TBP) is detected at ~3–16 FPKM in the sperm samples, whereas the strongly expressed glyceraldehyde-3-phosphate-dehydrogenase (GAPDH) gene reveals an expression value between ~600 and 1200 FPKM (Figure S3). The transcriptome analysis results in ~17,000 genes expressed in each sample (FPKM > 0.1 out of all ~23,000 genes). Next, we checked the transcriptomes for RNAs that were typically and specifically expressed in human spermatozoa (Sendler et al., 2013) to validate the quality of each dataset (Figure S4).

**Table 1:** **Sequencing details of the RNA-Seq datasets**.

**Sample**	**Library preparation**	**Read length [nt]**	**Read structure**	**Total prepared reads [million]**	**Reads with at least one reported alignment [%]**
Sperm donor 1	mRNA	75	Single	25.7	83.1
Sperm donor 2	mRNA	101	Paired-end	15.9	Left: 75.6 right: 77.9
Sperm donor 3-1	mRNA	101	Paired-end	10.8	Left: 81.3 right: 83.1
Sperm donor 3-2	mRNA	101	Paired-end	14.9	Left:83.1 right:85.2
Sperm donor 3-3	mRNA	101	Paired-end	12.6	Left: 75.2 right:75.8
Sperm donor 3-4	mRNA	101	Paired-end	12.7	Left: 78.2 right: 80.4
Sperm donor 4-1	mRNA	101	Paired-end	13.2	Left: 75.2 right: 77.6
Sperm donor 4-2	mRNA	101	Paired-end	13.5	Left: 79.9 right:82.3
Sperm donor 4-3	mRNA	101	Paired-end	13.8	Left: 76.1 right: 78.3
Sperm donor 4-4	mRNA	101	Paired-end	12.4	Left: 76.7 right: 78.9
Sperm donor 1-S	Stranded mRNA	101	Paired-end	14.5	Left: 77.6 right: 78.2
Sperm donor 4-S	Stranded mRNA	101	Paired-end	15.6	Left: 71.3 right: 72.1
Sperm donor 5-S	Stranded mRNA	101	Paired-end	14.5	Left: 80.7 right: 80.8

The transcripts of typical sperm-associated genes were expressed at high levels in all spermatozoa sample sets investigated. As an overview of the FPKM values for the expression of all genes, a histogram of the FPKM value distribution for Sperm sample 3–2 was calculated (Figure S5). The transcript structures were analyzed by visualizing the read alignment with the stranded RNA-Seq data sets using the IGV (Thorvaldsdóttir et al., 2013).

### Expression analysis of olfactory receptors

**Figure 1:**
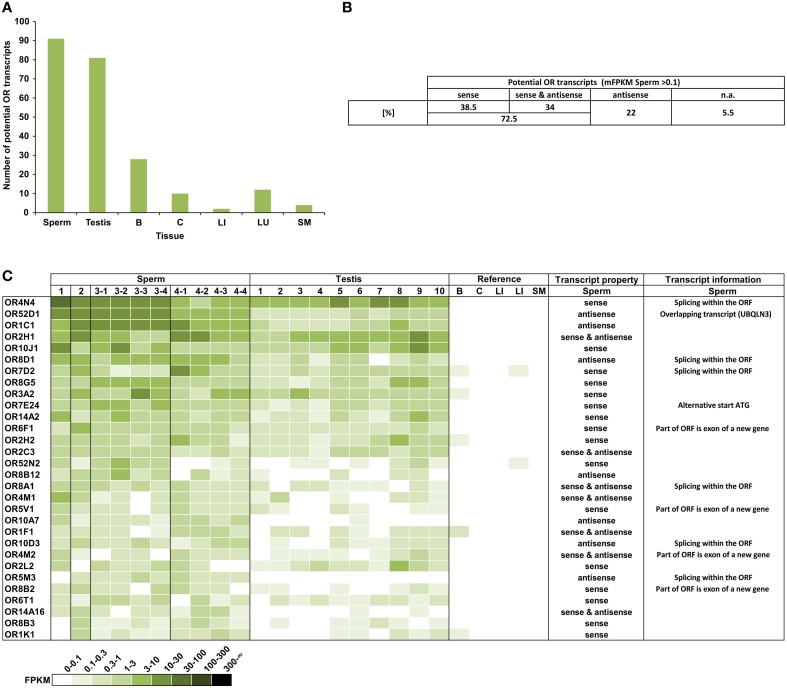
**A large number of different potential OR transcripts could be detected in human spermatozoa**. **(A)** Each bar represents the number of potential OR transcripts that is expressed with an FPKM > 0.1 in the samples investigated. For sperm and testis, the numbers of mFPKM > 0.1 are shown. The largest numbers of ORs were detected in the spermatozoa and testis. **(B)** The orientation of the detected potential OR transcripts. All transcripts with mFPKM > 0.1 were analyzed. n.a.: not available in the stranded RNA-Seq data sets. **(C)** The heat map shows FPKM values for the 30 most abundant potential OR transcripts found in the spermatozoa samples compared to the testis and reference tissues samples. Deeper colors indicate higher FPKM values and white indicates the absence of detectable transcripts. The ORs were sorted according to the mFPKM values of all spermatozoa samples. B, brain; C, colon; LI, liver; LU, lung; and SM, skeletal muscle. The transcripts property indicates the orientation of the detected transcripts in sperm samples relative to the OR transcripts. The transcript information indicates additional specifications of the detected transcripts in spermatozoa.

We analyzed the expression of OR transcripts in the human spermatozoa samples compared to the testis and reference tissue samples (Figure 1). Out of the 387 annotated OR genes, we detected 91 different potential OR transcripts in the human sperm samples (mean FPKM = mFPKM > 0.1; Figure 1A); the number of expressed transcripts in the testis is comparable (81 transcripts), but it is extremely lower in the reference tissues (brain, colon, liver, lung, and skeletal muscle; 2–28 transcripts).

In detail, we detected 50–108 different potential OR transcripts per sperm sample and 55–114 per testis sample. We detected 14 potential OR transcripts with the highest expression rate in all sperm samples investigated, indicating a coherent expression pattern of the highest transcripts in all RNA samples from the four donors. In total 37 potential OR transcripts were detected in all sperm samples and 118 were detected in at least two different sperm samples (FPKM > 0.1). We have previously detected a large number of the same OR transcripts in different testis samples (Flegel et al., 2013). Out of the 91 identified putative OR transcripts in human sperm (mFPKM > 0.1), 69% were also present in the analyzed testis samples (mFPKM > 0.1). When the low expressing OR transcripts were also taken into account (mFPKM < 0.1), we detected 207 putative OR transcripts in human sperm, and 87% of them were also traceable in the testis samples investigated. The plotted expression pattern for the mFPRM values of all OR transcripts in the sperm and testis samples is shown in Figure S6. The OR expression values of the testis and sperm samples are correlated (Pearson's correlation coefficient *r* = 0.86).

In addition to the normal mRNA-Seq data sets, we investigated stranded mRNA-Seq data sets from the human spermatozoa samples, which allowed us to investigate the orientation of the detected transcripts (Figure 1B). The analysis revealed that a certain number of transcripts are located in an antisense orientation relative to the OR transcripts. Twenty-two percent of the 91 putative OR transcripts (mFPKM > 0.1) have an antisense orientation, whereas we detected sense as well as antisense transcripts for 34% of the OR genes. Figure 1C visualizes the OR expression pattern sorted by the mFPKM value for all of the sperm samples. The 30 most abundant transcripts are displayed in comparison to the testis samples and the additional reference tissues. The spermatozoal transcript properties and additional transcript information were shown in detail for the 30 most highly expressed OR genes. The majority of potential OR transcripts that were detected in the spermatozoa and testis are absent in the reference tissues. Based on the FPKM values, the RNA expression for most of these transcripts is higher than in any other tissue investigated and even exceeds the expression of the housekeeping genes TBP and ß-Glucuronidase (GUSB). The FPKM values of 45 different potential OR transcripts were higher than 1 in at least one sperm sample (Figure S1).

Using the IGV, a detailed analysis of the mapped reads of the mRNA-Seq as well as the stranded RNA-Seq data for the most highly expressed OR genes was performed. For the four most highly expressed transcripts in the sense orientation, we analyzed the sequencing results and detected the presence of up to six non-translated upstream exons in addition to the coding exon (Figure S7). With the exception of OR4M2 and OR2B6, the read distribution of the 30 most highly expressed transcripts with the sense orientation support the expression of an OR transcript. In the case of OR6V1, OR4M2, and OR2B6, the aligned reads originate partially from a non-OR transcript with an exon partially overlapping the OR coding sequence (CDS).

**Figure 2:**
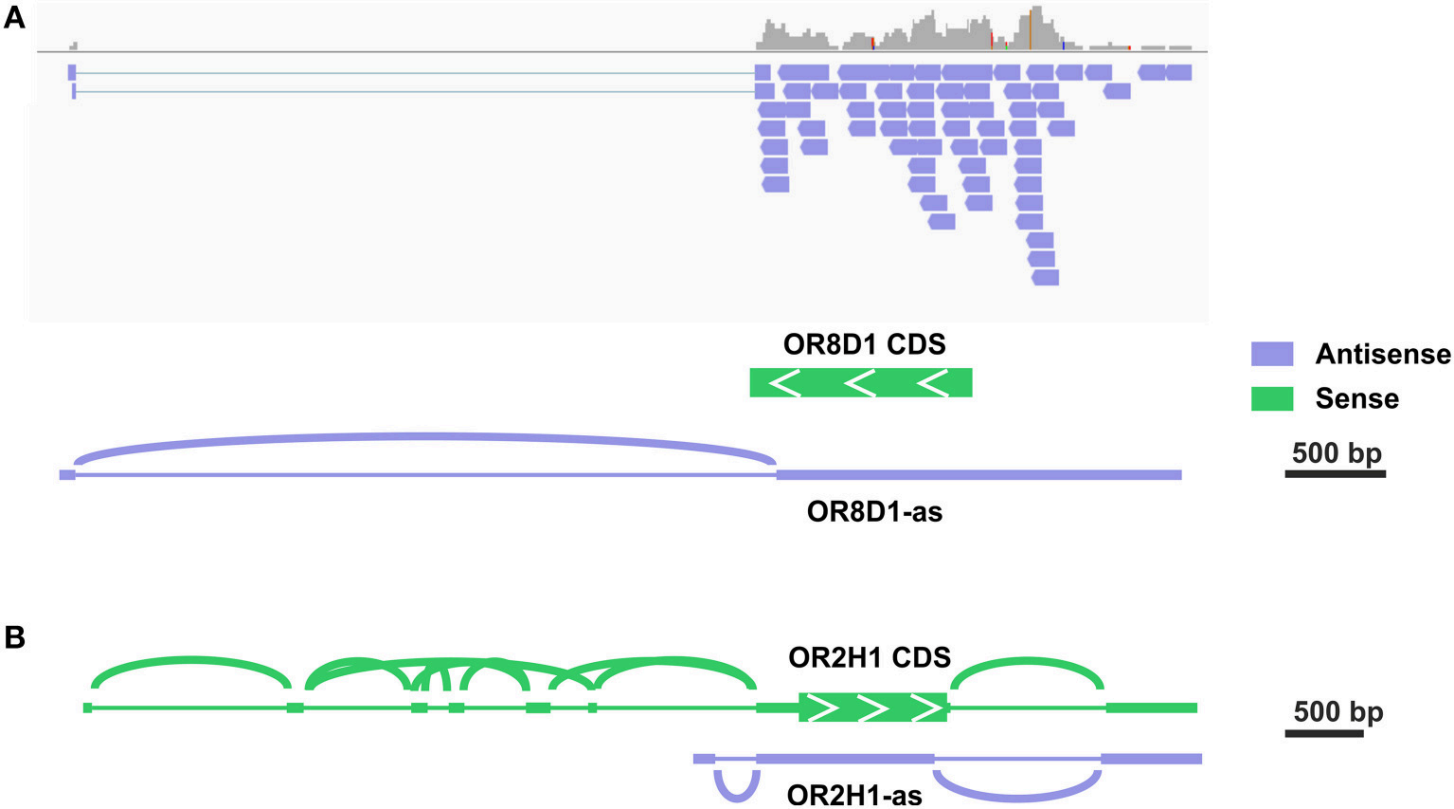
**Some potential OR transcripts are identified as antisense transcripts**. **(A)** Detailed analysis in the IGV of the stranded RNA-Seq data revealed the expression of antisense OR transcripts. The exemplary visualization of aligned reads to the OR8D1 gene is shown. All aligned reads originate from the RNA in antisense orientation relative to the OR8D1 transcript. **(B)** For OR2H1 sense as well as a low amount of antisense transcripts.

Out of the top 30 group, 7 transcripts were exclusively antisense in the stranded sequencing data relative to the expected OR transcript. In the case of OR52D1, this was due to an overlapping 3′ exon of the UBQLN3 transcript. However, in other cases we detected a novel transcript type that overlapped most of the OR coding sequence in the antisense orientation. For five of these antisense transcripts, the read coverage was high enough to assemble the transcript and to identify more than one exon (Figure S8). For example, the analysis for the putative OR1C1 transcript revealed a sperm-specific antisense transcript relative to the OR transcript consisting of 3–4 exons, which we named OR1C1-as (Figure S8A). For OR8D1, we also detected antisense transcripts (Figure 2). Figure 2A exemplarily visualizes the detected antisense transcript for OR8D1 using the stranded RNA-Seq data, indicating that the transcripts do not contain the OR coding sequence. The transcript would not code for the OR8D1 protein. A detailed analysis of the RNA-Seq data showed also a sperm-specific OR8D1-antisense transcript, which consists of 4–6 exons (OR8D1-as). Exon 5 of this sperm-specific transcript partially overlaps with the coding sequence of OR8D1. Further variability is generated by internal splicing in exon 5 (Figure S8B). All of the detected transcripts share a conserved exon intron structure and consist of a large exon covering ~90% of the OR coding sequence in antisense orientation. In addition, up to 5 smaller 5′ exons were found. However, the large exon never overlaps with 3′ end of the OR CDS. Relative to the OR CDS, the position of the splice acceptor site of the large exon is conserved and lies between 54 and 93 nt upstream of the stop codon in the region coding for the first amino acids C-terminally of TM7 of the OR (Figure S8G).

Three different ORs (OR1D2, OR7A5, and OR4D1) have already been characterized in human spermatozoa, although the expression of the OR transcripts was only shown in the testis (Spehr et al., 2003; Veitinger et al., 2011). In the present study, OR1D2 transcripts were identified in human spermatozoa (up to 0.61 FPKM) and testis (up to 0.24 FPKM). Furthermore, we confirmed the expression of OR7A5 and OR4D1 in sperm (OR7A5: up to 0.3 FPKM; OR4D1: up to 0.22 FPKM) and testis (OR7A5: up to 2.61 FPKM; OR4D1: up to 0.24 FPKM). We detected sense as well as antisense transcripts for all three ORs.

Previous studies described the expression of MHC-linked ORs in the testis, and these genes are localized within a cluster on the short arm of chromosome 6 (Ziegler et al., 2002; Volz et al., 2003; Flegel et al., 2013). MHC-linked ORs have been postulated as receptors for the detection of MHC peptides in testis and spermatozoa (Ziegler et al., 2002). In total, we detected 13 of 14 MHC-linked OR-genes in at least one sperm sample investigated, indicating a preferential expression in human spermatozoa. A complex framework of 5′ UTR exons was previously described for the MHC-linked OR transcripts (Volz et al., 2003). For OR2H1 (aka hs6M1-16), the most highly expressed MHC-linked OR transcript, the same was found in our RNA-seq data (Figure 2B, Figure S7D). Furthermore, we detected some antisense transcripts (OR2H1-as, Figure S8F). For four of the putative MHC-linked transcripts, only transcripts in antisense orientation relative to the respective OR transcript (OR2J1, OR14J1, OR12D2, and OR10C1) were detected.

### OR protein localization in human spermatozoa

**Figure 3:**
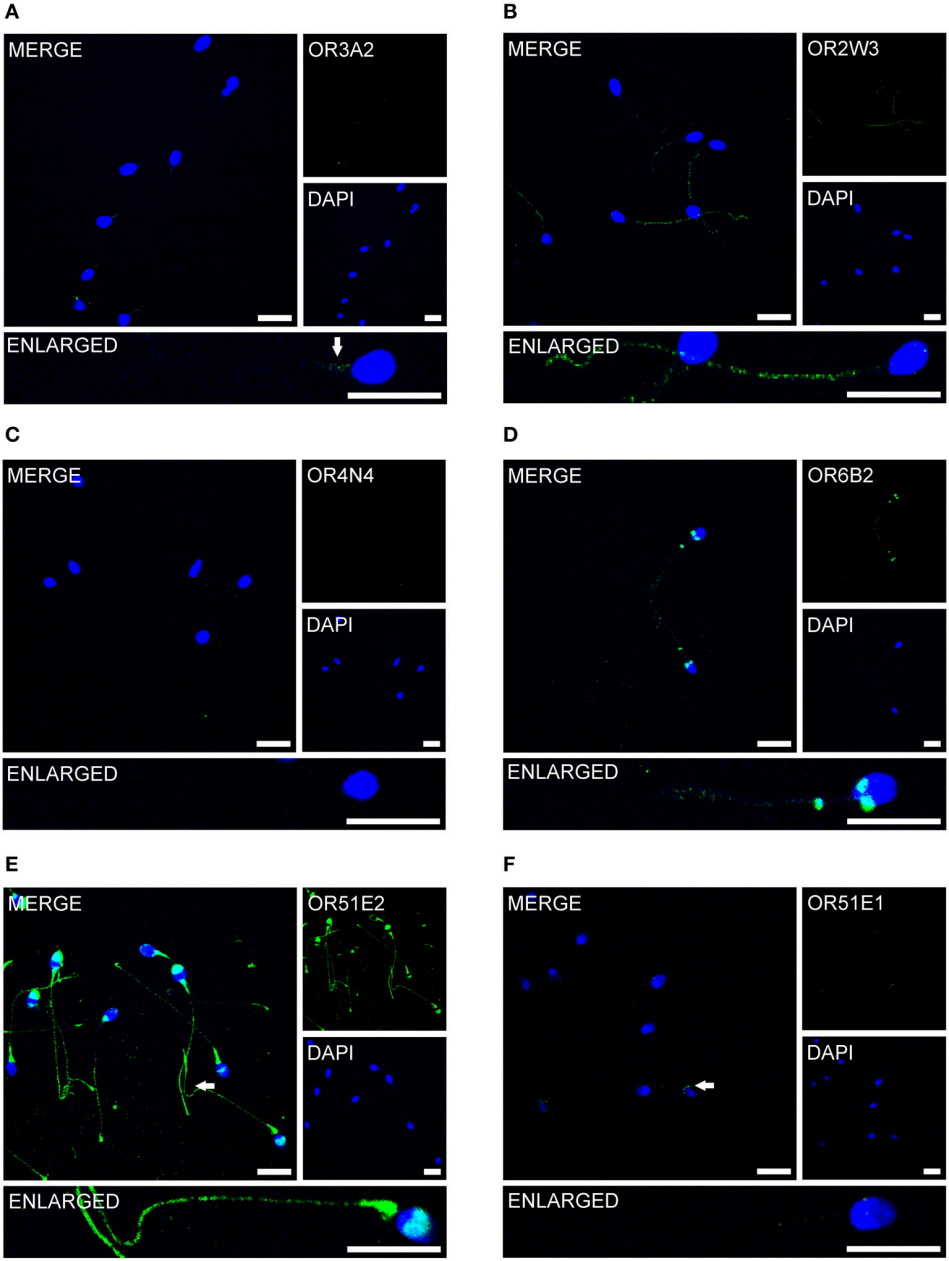
**OR proteins are localized to different compartments of human spermatozoa**. Immunocytochemical staining of human spermatozoa with different α-OR antibodies (green) revealed OR protein expression in different regions of human spermatozoa. DAPI staining (blue) was used to determine the number and location of the cell nuclei. The secondary antibody alone did not produce any non-specific staining (Figure S11). Scale bars: 10 μm. Immunocytochemical staining of human spermatozoa with the **(A)** α-OR2W3-antibody; **(B)** α-OR3A2-antibody; **(C)** α-OR4N4-antibody; **(D)** α-OR6B2-antibody; **(E)** α-OR51E2-antibody; and **(F)** α-O51E1-antibody are shown. The arrows indicate labeled structures.

**Figure 4:**
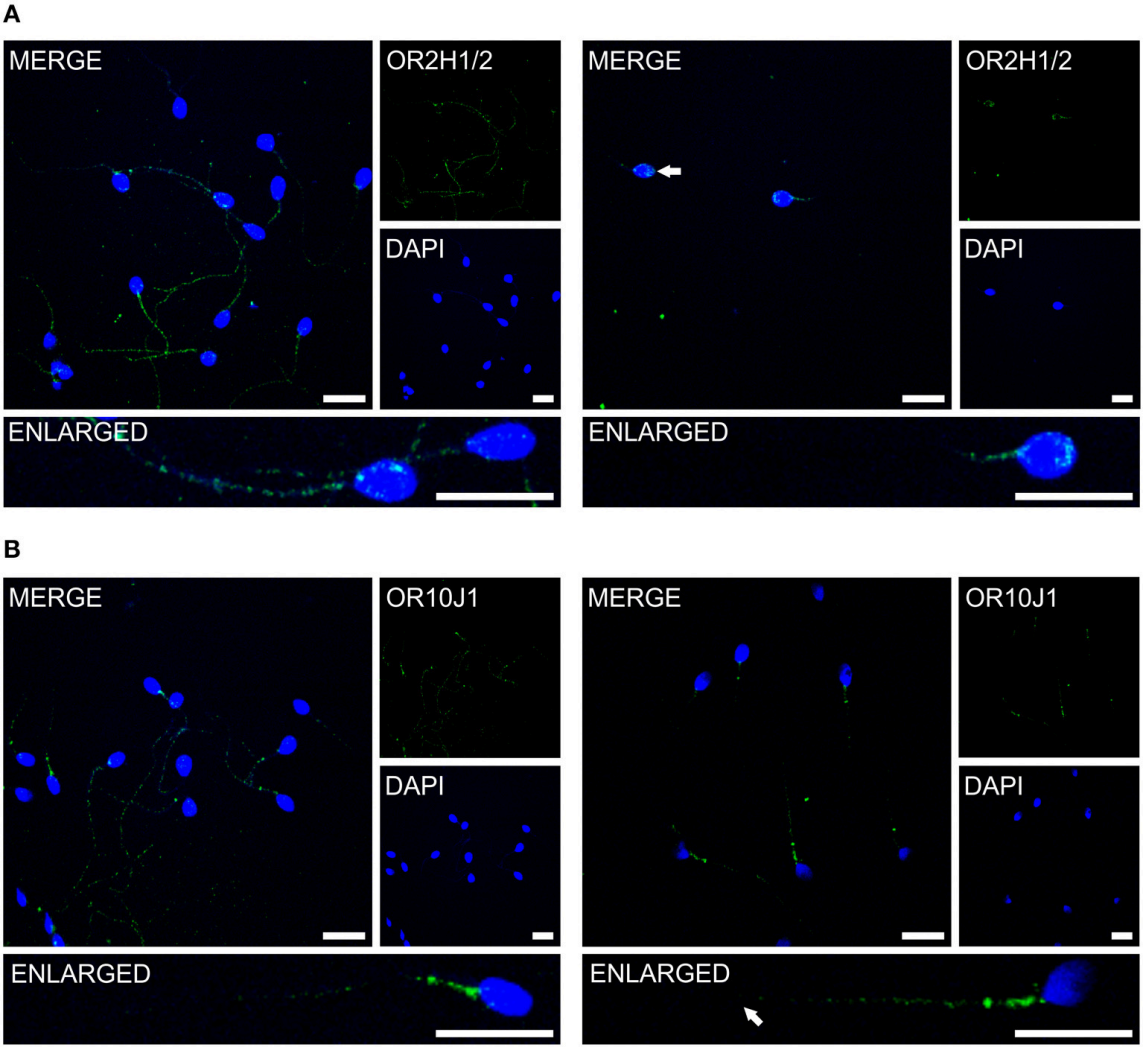
**The OR proteins OR2H1/2 and OR10J1 are localized to different compartments of human spermatozoa**. Immunocytochemical staining of human spermatozoa with the α-OR2H1 and the α-OR10J1 antibodies (green) revealed OR protein expression in different regions of human spermatozoa. DAPI staining (blue) was used to determine the number and location of the cell nuclei. Scale bars: 10 μm. **(A)** Immunocytochemical staining of OR2H1/2 in human spermatozoa. Images with a different focus (left, flagellum; right, head) are shown. **(B)** Immunocytochemical staining of OR10J1 in human spermatozoa. Images with a different focus (left, flagellum and midpiece; right, midpiece) are shown. The arrows indicate the labeled structures.

Previous immunocytochemistry studies revealed that ORs were localized at the base of flagella and in the flagellar midpieces of mature dog (Vanderhaeghen et al., 1993) and rat (Walensky et al., 1995) spermatozoa. The human OR1D2 protein has been localized to the midpiece (Neuhaus et al., 2006). To investigate the OR expression at the protein level, we also performed immunocytochemical staining with human spermatozoa. The localization of OR proteins in different regions may indicate a specific function. The specificity of 8 OR antibodies (α-OR4N4, α-OR10J1, α-OR2H1/2, α-OR2W3, α-OR3A2, α-OR51E1, α-OR51E2, α-OR6B2) was verified using recombinantly expressed rho-tagged ORs in Hana3A cells (Figures S9, S10). The panel of specific antibodies includes those for ORs with low, moderate and high mRNA expression in human sperm. Subsequent immunocytochemical staining of human spermatozoa from at least four different donors revealed a consistent expression pattern of several OR proteins in different parts of human sperm. The OR3A2 protein was detected in the midpiece of human sperm (Figure 3A). OR2W3 was present in the flagella of human spermatozoa (Figure 3B). Although specificity for the α-OR4N4 antibody was verified with recombinantly expressed OR4N4 protein in Hana3A cells, we were not able to detect the OR4N4 protein in human spermatozoa from four different donors (Figure 3C). The OR6B2 protein was localized to the equatorial segment in the head and the flagellar principal piece of human sperm (Figure 3D). The OR51E2 protein was identified in the flagella, the midpiece, and on the acrosomal cap (Figure 3E). However, OR51E2 was not present in all of the acrosomal caps of each spermatozoa investigated. Low amounts of the OR51E1 protein were detected in ~25% of the sperm heads and were located in the region of the acrosomal cap (Figure 3F). α-OR2H1/2 antibody staining revealed the OR2H1/2 protein on the head and the flagella of human spermatozoa (Figure 4A). α-OR10J1 antibody staining revealed a weak punctate staining in the flagella of sperm. Strong punctuate staining was detected 3 μm caudal to the midpiece (annulus) (Figure 4B).

### Identification of three new OR-ligand pairs

As previously described, OR2W3 is a broadly expressed OR (Flegel et al., 2013), and we could show that the OR2W3 transcript and protein were detected in human spermatozoa in the current study. OR2H1 and OR10J1 showed high and specific transcript and protein expression in human spermatozoa. The activating odorants for all three receptors are unknown. Therefore, we expressed these three recombinant ORs in Hana3A cells (Saito et al., 2004) and screened with different odorant mixtures using the previously described calcium imaging technique (Wetzel et al., 1999; Spehr et al., 2003; Neuhaus et al., 2009; Veitinger et al., 2011; Busse et al., 2014). The deorphanization of OR2W3, OR2H1, and OR10J1 revealed significant activation by the specific odorants nerol, methional, and dimetol, respectively (Figure S12). Mock-transfected Hana3A cells showed no specific odorant-induced Ca^2+^ signals for any of the tested odorant (Figure S12). Together, we revealed the ligands for three ORs, which were newly identified at the transcript and protein level in human sperm.

### Structurally diverse OR ligands induce Ca^2+^ signals in human spermatozoa

The change in the intracellular calcium concentration is an important mediator of physiological processes in spermatozoa (Darszon et al., 2011). Furthermore, the activation of the previously characterized ORs induced Ca^2+^ signals in spermatozoa (Spehr et al., 2003; Fukuda et al., 2004; Veitinger et al., 2011). Consequently, we investigated if the newly identified agonists of OR2W3, OR2H1, and OR10J1 were able to induce Ca^2+^ signals in human spermatozoa. Furthermore, we performed a literature search to determine whether further ligands for the newly detected spermatozoal ORs are known. The following 10 odorants were tested: bourgeonal (OR1D2; Spehr et al., 2003), coumarin (OR1C1 and OR2J2; Adipietro et al., 2012), dimetol (OR10J1, this study), methional (OR2H1, this study), myrac (OR7A5; Veitinger et al., 2011), ß ionone (OR51E2; Neuhaus et al., 2009), nerol (OR2W3; this study), nonanoic acid (OR51E1; Saito et al., 2009), sotolone (OR8D1; Adipietro et al., 2012), and methyl octanoate (OR52D1; Sanz et al., 2005).

**Figure 5:**
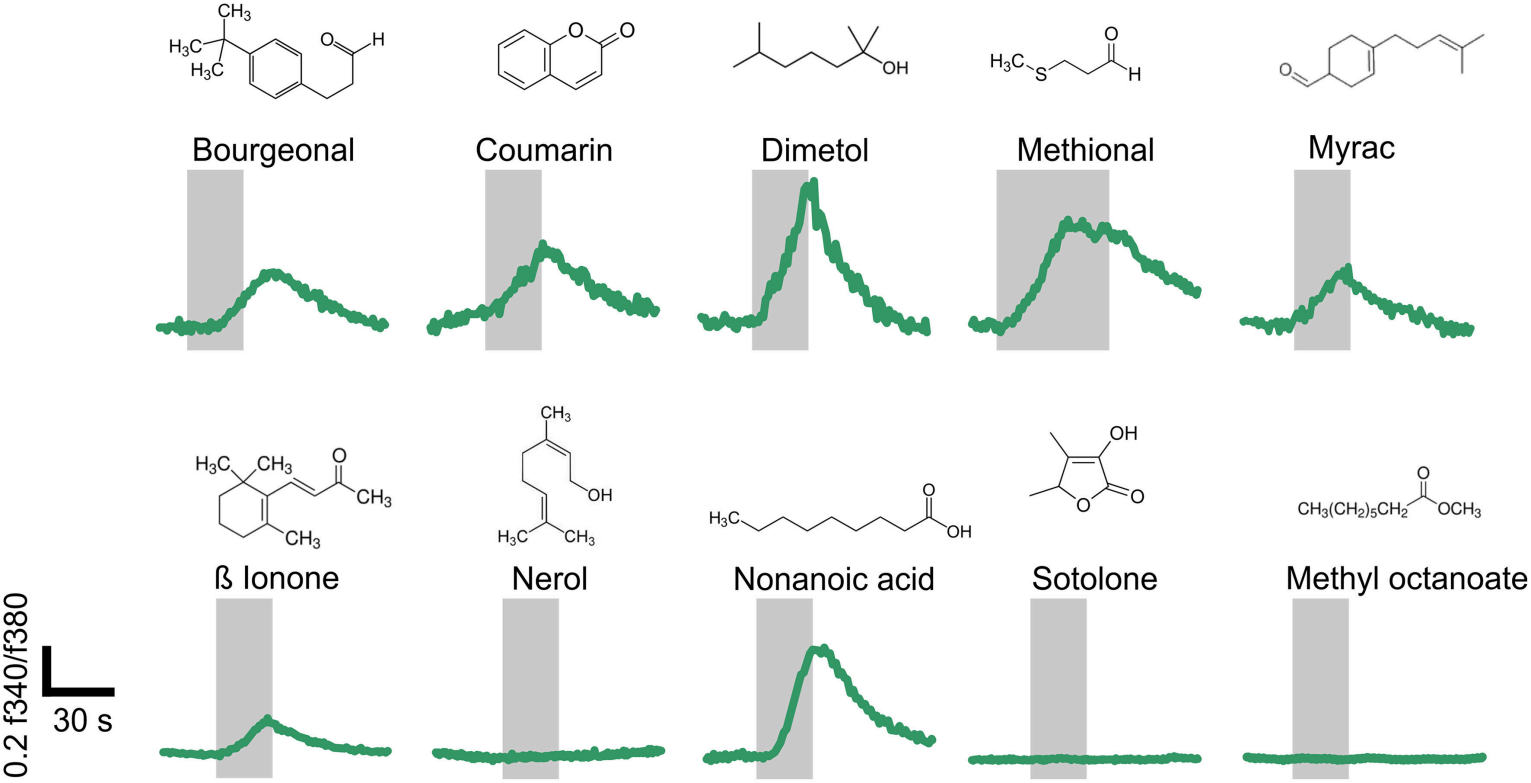
**Structurally diverse odorants evoke Ca^**2+**^ signals in human spermatozoa**. Representative calcium imaging traces from odorant-stimulated human spermatozoa are shown. Seven of the ten different odorants (100 μM; Nonanoic acid: 300 μM) induced transient Ca^2+^ signals in human spermatozoa upon odorant stimulation for 20–40 s. The cytosolic Ca^2+^ levels are monitored as the integrated f340/f380 fluorescence ratio expressed as a function of time. The gray bars indicate the stimulus duration. The odorants were tested in at least three different experiments.

Using single-cell calcium imaging experiments, we monitored the changes in intracellular calcium concentration in human sperm upon short term (20–40 s) odorant stimulation (100–300 μM) (Figure 5). Seven of the ten tested OR ligands transiently induced Ca^2+^ signals in the majority of all vital spermatozoa (65–93%). Progesterone was used as a vitality control stimulus (500 nM) at the end of each measurement (data not shown). Three of the ten tested odorants did not induce any Ca^2+^ signals in human sperm, even when higher odorant concentrations (500 μM) were applied (data not shown). Interestingly, two of these odorants are ligands for the ORs for which we only detected antisense transcripts.

Next, we investigated which calcium channels may be involved in the odorant-induced Ca^2+^ signals. Four different calcium channel families have been attributed a role in human sperm calcium signaling. In previous studies, the mRNAs coding for these calcium channels have been isolated from either spermatogenic cells or mature sperm, and the presence of the corresponding proteins has been confirmed (reviewed in Darszon et al., 2011). We confirmed the expression of a variety of calcium channels that are potentially involved in the odorant-induced Ca^2+^ signals (Figures S13–S16). Of the voltage-gated CatSper calcium channels, we detected the four different α-like subunits (CATSPER1-4) with mFPKM values of 4.4 (CATSPER1), 7 (CATSPER2), 12.1 (CATSPER3), and 3.3 (CATSPER4), as well as the auxiliary subunits (CATSPERB-G) with mFPKM values of 1.9 (CATSPERB), 8.2 (CATSPERD), and 72.3 (CATSPERG) (Figure S13). Within the TRP channel family, TRPV6 (12.7 mFPKM) and TRPC1 (3.4 mFPKM) showed the highest and most consistent expression in all sperm samples investigated (Figure S14). For the voltage-dependent calcium channels, we detected the highest expression for CACNA1H (T type; 91.3 mFPKM), CACNA1B (N type; 19.8 mFPKM), and CACNA1C (L type; 4.9 mFPKM) (Figure S15). The olfactory CNG channel subunit A4 (5.3 mFPKM) and the rod CNG channel subunit B3 (17.3 mFPKM) were consistently expressed in all spermatozoa samples (Figure S16).

To determine if the odorant-induced changes in Ca^2+^ concentration originate from intracellular or extracellular Ca^2+^ sources, we performed calcium imaging experiments with human spermatozoa under calcium-free conditions (10 mM EGTA). As shown in Figure S17A, the absence of extracellular Ca^2+^ completely abolished the effect of dimetol and nonanoic acid. The measurements showed that the odorant-evoked Ca^2+^ signals of human spermatozoa depend on extracellular calcium. To investigate whether a calcium channel is involved in the odorant-induced Ca^2+^ signals, calcium imaging studies were performed using calcium channel blockers. The co-application of the non-selective calcium channel blocker mibefradil (30 μM) (Clozel et al., 1997; Wennemuth et al., 2000; Strünker et al., 2011) significantly diminished the odorant-induced Ca^2+^ signals of all odorants tested (coumarin, methional, dimetol, and nonanoic acid) (Figure S17B). The amplitudes of the odorant-evoked Ca^2+^ signals were suppressed by ~95%, indicating the involvement of calcium channels, such as T-type calcium or CatSper channels. The amplitudes of the dimetol-induced Ca^2+^ signals were only suppressed by 35%. The odorant-induced Ca^2+^ signals recovered after mibefradil was washed out.

## Discussion

It is hypothesized that spermatozoa possess chemosensory properties to respond to a large number of chemical cues in the female genital tract during their passage toward the egg. Studies indicate that capacitated spermatozoa are guided from the storage site of the oviduct to the egg primarily by a combination of chemotaxis (Cohen-Dayag et al., 1995; Sun et al., 2005) and thermotaxis (Bahat et al., 2003), and may be assisted by oviductal contractions (Battalia and Yanagimachi, 1979; Eisenbach and Giojalas, 2006). The complete spermatozoal repertoire of potential chemoreceptor transcripts and proteins is largely unknown. In this study, we analyzed the broad expression profile of OR transcripts and proteins in human spermatozoa and investigated the physiological effects of known OR ligands in human spermatozoa.

### The olfactory transcriptome in human spermatozoa

Up to date, a comprehensive expression analysis of OR transcripts in mammalian spermatozoa was lacking. Spermatozoal RNA reflects the significant proportion of the RNA synthesized prior to transcriptional arrest that is stored in a stable form in preparation for its translation during the later stages of terminal spermatogenic differentiation (Steger, 2001). Transcripts that are much more highly expressed may indicate a final burst of transcriptional activity during spermatid differentiation (Sendler et al., 2013). Therefore, many of the present spermatozoal RNAs are considered remnants. However, spermatozoa also transmit RNA to the oocyte at fertilization (Ostermeier et al., 2004), but their functional significance remains to be established.

In the present study, the first olfactory transcriptome analysis of human spermatozoa revealed the expression of a large panel of about 90 putative OR transcripts. The majority of the detected OR transcripts were newly identified in human spermatozoa in our study. Human spermatozoa and testes expressed the highest number of different putative OR transcripts compared to the reference tissues investigated (brain, colon, liver, lung, skeletal muscle). Additionally, we detected a strong overlapping OR expression pattern for spermatozoa and testis (up to ~90%), demonstrating that most of the putative OR transcripts detected in the testis are derived from spermatozoa or precursor cells and not from Leydig or Sertoli cells. Interestingly, most of the detected OR transcripts were exclusively found in human sperm and testis and not in the reference tissues, indicating a specialized function in sperm. ORs are specialized to detect a broad variety of chemical substances, and the current RNA-Seq dataset raised the question of why such a huge number of OR transcripts is present in mature human spermatozoa.

One fifth of the detected potential OR transcripts are exclusively antisense relative to the respective OR transcripts. For six transcripts the read coverage was sufficient to detect a conserved exon intron structure and a large exon covering ~90% of the OR coding sequence in antisense orientation. Relative to the OR CDS, the position of the splice acceptor site of the large exon is conserved. Due to this conserved features, these transcripts form a novel family of transcripts specifically expressed in sperm and testis and were not found in the recently investigated reference tissues (Flegel et al., 2013). All transcrpis were antisense to class II ORs, however from different subfamilies. The function of these antisense transcripts is still completely unknown. However, there is evidence that antisense transcripts can be transferred into the oocyte and can influence early embryogenesis (Hosken and Hodgson, 2014). In general, antisense transcripts can act as regulatory elements involved in transcription modulation hybridization of sense-antisense RNA partners, and chromatin modification (Mills et al., 2013).

### OR proteins in human spermatozoa

**Figure 6:**
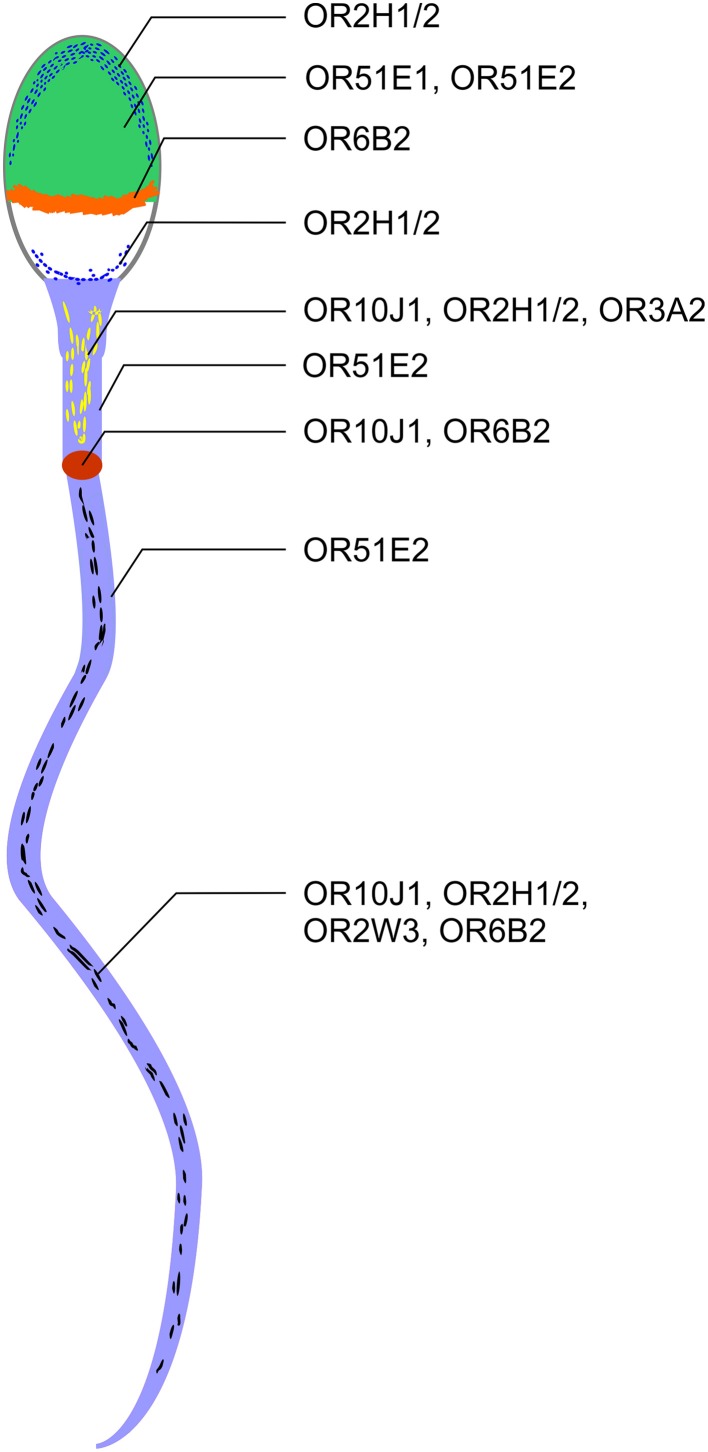
**ORs are localized to different regions in human spermatozoa**. OR2H1/2, OR51E1, and OR51E2 were detected on the acrosome cap (green). OR2H1/2 was also detectable in the caudal part of the sperm head (blue line). OR6B2 was localized to the equatorial segment (orange). OR51E2, OR10J1, OR2H1/2, and OR3A2 are localized to the midpiece (yellow dashes). OR10J1 and OR6B2 proteins were detected at a particular position on the beginning of the flagella (annulus, red oval). Strong staining with the OR51E2 antibody was detected in the entire flagella (light blue). OR10J1, OR2H1/2, OR2W3, and OR6B2 showed a punctate distribution in the flagella of human spermatozoa (black dashes).

Using immunocytochemistry, we determined the compartment-specific localization of different OR proteins in human spermatozoa (Figure 6) and localized them to the tail, the midpiece and the head of human sperm. The majority of OR proteins were detected in all human spermatozoa, indicating that one sperm expresses multiple ORs, as already postulated by Veitinger et al. (2011). Furthermore, the co-localization of different OR transcripts was shown in murine round spermatids (Fukuda and Touhara, 2006).

OR proteins are localized at specialized cellular compartments, such as the equatorial segment, the midpiece, and the tail. For example, we detected a strong and defined OR6B2-staining in the equatorial segment which suggests a role for OR6B2 during acrosomal exocytosis and sperm-egg binding (Wolkowicz et al., 2008). Unfortunately, the ligands for OR6B2 are unknown. OR51E2-positive acrosomal caps were only detected in some sperm. For OR51E1, we determined only a weak staining in the head of ~25% of the sperm investigated. It is conceivable that the OR protein level decreased upon acrosomal exocytosis.

We observed no correlation between the amount of the detected transcripts and the intensity of the protein staining. For example, we detected a strong staining for OR51E2 protein, although OR51E2 does not belong to the 30 most highly expressed OR transcripts. We detected highly abundant OR4N4 transcripts in all spermatozoa and testis samples, however, were not able to detect the OR4N4 protein in human spermatozoa (Figure 3) or testis (data not shown). Nevertheless, the results of the present study showed, for the first time, that the OR proteins are localized to different human sperm regions.

### The physiological role of ORs in human spermatozoa

In previous studies, we showed that OR1D2 can be activated by the odorant bourgeonal (Spehr et al., 2003). OR1D2 is localized to the midpiece of human spermatozoa (Neuhaus et al., 2006). OR4D1 and OR7A5 are expressed in human testis and sperm and they can be activated by naturally occurring ligands (OR4D1: 5a-androst-16-en-3-one; OR7A5: 4-hydroxy-2,5-dimethyl-3(2H)-furanone) (Hartmann et al., 2013). For all three ORs, their activations were linked to a chemokinetic function in human spermatozoa (Veitinger et al., 2011). However, odorants were also suggested to directly affect the CatSper calcium channel in human spermatozoa (Brenker et al., 2012).

In the present study, we investigated 14 ORs in spermatozoa, together with the previously characterized spermatozoal ORs, in more detail (Table 2). We were able to deorphanize three newly identified spermatozoal ORs (OR2W3-nerol; OR10J1-dimetol; OR2H1-methional). Interestingly, methional is an endogenously occurring ligand in the female reproductive tract (Hartmann et al., 2013).

**Table 2:** **Characterization of the ORs expressed in human spermatozoa**.

**OR**	**≤FPKM in sperm**	**Transcript properties**	**Ligand**	**References**	**Specific antibody**	**Protein**	**Ligand-induced Ca^2+^ signals in sperm**
OR10J1	14.4	Sense RNA	Dimetol	This study	✓	✓	✓
OR1C1	16.9	Antisense RNA	Coumarin	Adipietro et al., 2012	n.a.	n.a.	✓
OR1D2	0.6	Sense and antisense RNA	Bourgeonal	Spehr et al., 2003	Neuhaus et al., 2006 ✓	✓	Spehr et al., 2003 ✓
OR2H1	26.6	Sense RNA	Methional	This study	✓	✓	✓
OR2J2	0.5	Sense RNA	Coumarin	Adipietro et al., 2012	n.a.	n.a.	✓
OR2W3	0.9	Sense RNA	Nerol	This study	✓	✓	✗
OR3A2	10.5	Sense RNA	✗	n.a.	✓	✓	n.a.
OR4D1	0.2	Sense and antisense RNA	PI-23472	Veitinger et al., 2011	n.a.	n.a.	Veitinger et al., 2011 ✓
OR4N4	66.2	Sense RNA	✗	n.a.	✓	✗	n.a.
OR51E1	0.4	Sense RNA	Nonanoic acid	Saito et al., 2009	✓	✓	✓
OR51E2	0.8	Sense RNA	β Ionone	Neuhaus et al., 2009	✓	✓	✓
OR52D1	15.6	Antisense RNA	Methyl octanoate	Sanz et al., 2005	n.a.	n.a.	✗
OR6B2	0.2	Sense RNA	✗	n.a.	✓	✓	n.a.
OR7A5	0.3	Sense and antisense RNA	Myrac	Veitinger et al., 2011	n.a.	n.a.	Veitinger et al., 2011 ✓
OR8D1	8.3	Antisense RNA	Sotolone	Adipietro et al., 2012	n.a.	n.a.	✗

*n.a., not available; ✓, positive result; ✗, negative result*.

We investigated the effects of 10 OR ligands for ORs, which were detected at the RNA and/or protein level in human sperm, in single-cell calcium imaging experiments. Seven of the ten tested OR ligands induced Ca^2+^ signals in human spermatozoa. The effects of bourgeonal and myrac were already described in detail (Spehr et al., 2003; Veitinger et al., 2011). The ligands for OR8D1 and OR52D1 (sotolone and methyl octanoate) did not induce any Ca^2+^ signals. Interestingly, we only identified antisense transcripts for these ORs relative to the respective OR transcript. Nerol, a ligand of OR2W3, was the only case in which an OR ligand didn't induce any Ca^2+^ signal although expression of the respective receptor was detected at RNA and protein level.

We observed that odorant-induced Ca^2+^ signals strongly depend on the extracellular calcium that enters the cell via a calcium permeable channel. Mibefradil inhibits CatSper (Strünker et al., 2011) and also further calcium channels in sperm (e.g., CACNA1H) (Clozel et al., 1997; Wennemuth et al., 2000; Chiu et al., 2008; Bhandari et al., 2010). In our experiments, mibefradil blocked Ca^2+^ signals induced by all tested odorants except for those evoked by dimetol which were suppressed by only 35%. Dimetol is thus the only tested odorant that raises intracellular Ca^2+^ levels at least partly independent of mibefradil-sensitive channels.

The odorant-induced cascade in human sperm was previously shown to be independent of adenylyl cyclase activation and second messengers (cAMP and cGMP), but strongly depends on a calcium channels (Veitinger et al., 2011; Brenker et al., 2012). It was suggested that the CatSper channel is directly activated by odorants (Brenker et al., 2012). Whether all newly tested odorants also directly act on CatSper or trigger a signaling cascade by activating an OR remains elusive. A variety of mechanisms are possible including direct OR-triggered G protein activation of calcium channels (Dascal, 2001). Aside from G protein activation, PDZ domain-containing proteins, JAK/STATs, Src-family tyrosine kinases, and GRKs/beta-arrestins have been proposed to directly relay signals from GPCRs (Sun et al., 2007). Also for one OR, a G-protein independent mechanism was found. In LNCaP-cells, OR51E2 activates a Src kinase independently of a G protein which directly opens TRPV6 (Neuhaus et al., 2009; Spehr et al., 2011). Notably, we determined that TRPV6 was highly expressed in human spermatozoa (Figure S14).

Together, the odorant-induced signal transduction cascade in human spermatozoa, which induces a Ca^2+^ increase, is still elusive and remains to be solved. Particular ORs may be involved in different physiological processes, enabling spermatozoa to respond to a variety of cues on their way to the egg. The expression of a plethora of OR transcripts and proteins in different compartments of human sperm suggests that these receptors take part in physiological processes other than chemotaxis and chemokinesis. Perhaps only a small subset of ORs is involved in chemotaxis of spermatozoa, particularly those ORs that were detected at protein level on the flagella of human spermatozoa. It is conceivable that some ORs are involved in acrosomal exocytosis, capacitation, and spermatogenesis or epididymal maturation (Fukuda and Touhara, 2006).

## Conclusions

The results of the present study showed, for the first time, a complete expression analysis of the OR transcripts in spermatozoal RNA. We detected 91 putative OR transcripts, of which 72% consisted of at least partial sense transcripts. One fifth of the detected potential OR transcripts were antisense. We were able to localize seven different OR proteins in different compartments of human sperm. Ligands for several of ORs expressed in sperm induce intracellular Ca^2+^ signals involving mibefradil-sensitive Ca^2+^-channels. However, the functional involvement of the ORs in sperm physiology has to be resolved in the future.

## Author contributions

Wrote the paper: CF and GG; analyzed the data: CF, FV, AH, BS, SO, CB, MM, GW, JA and GG; designed the experiments: CF, GG, HH, NB and GW; conducted the experiments: CF, FV, AH, BS, SO and MM.

### Conflict of interest statement

The authors declare that the research was conducted in the absence of any commercial or financial relationships that could be construed as a potential conflict of interest.

## References

[B1] Adipietro K. A. Mainland J. D. Matsunami H. (2012). Functional evolution of mammalian odorant receptors. PLoS Genet. 8:e1002821. 10.1371/journal.pgen.1002821 22807691 PMC3395614

[B2] Bahat A. Tur-Kaspa I. Gakamsky A. Giojalas L. C. Breitbart H. Eisenbach M. (2003). Thermotaxis of mammalian sperm cells: a potential navigation mechanism in the female genital tract. Nat. Med. 9, 149–150. 10.1038/nm0203-149 12563318

[B3] Battalia D. E. Yanagimachi R. (1979). Enhanced and co-ordinated movement of the hamster oviduct during the periovulatory period. J. Reprod. Fertil. 56, 515–520. 10.1530/jrf.0.0560515 573324

[B4] Bhandari B. Bansal P. Talwar P. Gupta S. K. (2010). Delineation of downstream signalling components during acrosome reaction mediated by heat solubilized human zona pellucida. Reprod. Biol. Endocrinol. 8:7. 10.1186/1477-7827-8-7 20096131 PMC2832785

[B5] Brenker C. Goodwin N. Weyand I. Kashikar N. D. Naruse M.. (2012). The CatSper channel: a polymodal chemosensor in human sperm. EMBO J. 31, 1654–1665. 10.1038/emboj.2012.30 22354039 PMC3321208

[B6] Busse D. Kudella P. Grüning N. Gisselmann G. Ständer S. Luger T.. (2014). A synthetic sandalwood odorant induces wound healing processes in human keratinocytes via the olfactory receptor OR2AT4. J. Invest. Dermatol. 134, 2823–2832. 10.1038/jid.2014.273 24999593

[B7] Chiu P. C. N. Wong B. S. T. Chung M. Lam K. K. W. Pang R. T. K.. (2008). Effects of native human zona pellucida glycoproteins 3 and 4 on acrosome reaction and zona pellucida binding of human spermatozoa. Biol. Reprod. 79, 869–877. 10.1095/biolreprod.108.069344 18667750

[B8] Clozel J. P. Ertel E. A. Ertel S. I. (1997). Discovery and main pharmacological properties of mibefradil (Ro 40-5967), the first selective T-type calcium channel blocker. J. Hypertens. Suppl. 15, S17–S25. 10.1097/00004872-199715055-00004 9481612

[B9] Cohen-Dayag A. Tur-Kaspa I. Dor J. Mashiach S. Eisenbach M. (1995). Sperm capacitation in humans is transient and correlates with chemotactic responsiveness to follicular factors. Proc. Natl. Acad. Sci. U.S.A. 92, 11039–11043. 10.1073/pnas.92.24.11039 7479932 PMC40566

[B10] Darszon A. Nishigaki T. Beltran C. Treviño C. L. (2011). Calcium channels in the development, maturation, and function of spermatozoa. Physiol. Rev. 91, 1305–1355. 10.1152/physrev.00028.2010 22013213

[B11] Dascal N. (2001). Ion-channel regulation by G proteins. Trends Endocrinol. Metab. 12, 391–398. 10.1016/S1043-2760(01)00475-1 11595540

[B12] Eisenbach M. Giojalas L. C. (2006). Sperm guidance in mammals - an unpaved road to the egg. Nat. Rev. Mol. Cell Biol. 7, 276–285. 10.1038/nrm1893 16607290

[B13] Feldmesser E. Olender T. Khen M. Yanai I. Ophir R.. (2006). Widespread ectopic expression of olfactory receptor genes. BMC Genomics 7:121. 10.1186/1471-2164-7-121 16716209 PMC1508154

[B14] Firestein S. (2001). How the olfactory system makes sense of scents. Nature 413, 211–218. 10.1038/35093026 11557990

[B15] Flegel C. Manteniotis S. Osthold S. Hatt H. Gisselmann G. (2013). Expression profile of ectopic olfactory receptors determined by deep sequencing. PLoS ONE 8:e55368. 10.1371/journal.pone.0055368 23405139 PMC3566163

[B16] Fukuda N. Touhara K. (2006). Developmental expression patterns of testicular olfactory receptor genes during mouse spermatogenesis. Genes Cells 11, 71–81. 10.1111/j.1365-2443.2005.00915.x 16371133

[B17] Fukuda N. Yomogida K. Okabe M. Touhara K. (2004). Functional characterization of a mouse testicular olfactory receptor and its role in chemosensing and in regulation of sperm motility. J. Cell. Sci. 117(Pt 24), 5835–5845. 10.1242/jcs.01507 15522887

[B18] Glusman G. Yanai I. Rubin I. Lancet D. (2001). The complete human olfactory subgenome. Genome Res. 11, 685–702. 10.1101/gr.171001 11337468

[B19] Hartmann C. Triller A. Spehr M. Dittrich R. Hatt H. (2013). Sperm-activating odorous substances in human follicular fluid and vaginal secretion: identification by gas chromatography–olfactometry and Ca2+ imaging. ChemPlusChem 78, 695–702. 10.1002/cplu.201300008 31986625

[B20] Hosken D. J. Hodgson D. J. (2014). Why do sperm carry RNA? Relatedness, conflict, and control. Trends Ecol. Evol. (Amst.) 29, 451–455. 10.1016/j.tree.2014.05.006 24916312

[B21] Martínez-López P. Treviño C. L. de la Vega-Beltrán J. L. De Blas G. Monroy E. Beltrán C.. (2011). TRPM8 in mouse sperm detects temperature changes and may influence the acrosome reaction. J. Cell. Physiol. 226, 1620–1631. 10.1002/jcp.22493 21413020

[B22] Meyer D. Voigt A. Widmayer P. Borth H. Huebner S.. (2012). Expression of Tas1 taste receptors in mammalian spermatozoa: functional role of Tas1r1 in regulating basal Ca^2+^ and cAMP concentrations in spermatozoa. PLoS ONE 7:e32354. 10.1371/journal.pone.0032354 22427794 PMC3303551

[B23] Mills J. D. Kawahara Y. Janitz M. (2013). Strand-specific RNA-seq provides greater resolution of transcriptome profiling. Curr. Genomics 14, 173–181. 10.2174/1389202911314030003 24179440 PMC3664467

[B24] Mortazavi A. Williams B. A. McCue K. Schaeffer L. Wold B. (2008). Mapping and quantifying mammalian transcriptomes by RNA-Seq. Nat. Methods 5, 621–628. 10.1038/nmeth.1226 18516045 PMC13303166

[B25] Neuhaus E. M. Mashukova A. Barbour J. Wolters D. Hatt H. (2006). Novel function of beta-arrestin2 in the nucleus of mature spermatozoa. J. Cell. Sci. 119(Pt 15), 3047–3056. 10.1242/jcs.03046 16820410

[B26] Neuhaus E. M. Zhang W. Gelis L. Deng Y. Noldus J.. (2009). Activation of an olfactory receptor inhibits proliferation of prostate cancer cells. J. Biol. Chem. 284, 16218–16225. 10.1074/jbc.M109.012096 19389702 PMC2713531

[B27] Ostermeier G. C. Goodrich R. J. Moldenhauer J. S. Diamond M. P. Krawetz S. A. (2005). A suite of novel human spermatozoal RNAs. J. Androl. 26, 70–74. 10.1002/j.1939-4640.2005.tb02874.x 15611569

[B28] Ostermeier G. C. Miller D. Huntriss J. D. Diamond M. P. Krawetz S. A. (2004). Reproductive biology: delivering spermatozoan RNA to the oocyte. Nature 429:154. 10.1038/429154a 15141202

[B29] Parmentier M. Libert F. Schurmans S. Schiffmann S. Lefort A.. (1992). Expression of members of the putative olfactory receptor gene family in mammalian germ cells. Nature 355, 453–455. 10.1038/355453a0 1370859

[B30] Riffell J. A. Krug P. J. Zimmer R. K. (2002). Fertilization in the sea: the chemical identity of an abalone sperm attractant. J. Exp. Biol. 205(Pt 10), 1439–1450. 11976355 10.1242/jeb.205.10.1439

[B31] Saito H. Chi Q. Zhuang H. Matsunami H. Mainland J. D. (2009). Odor coding by a Mammalian receptor repertoire. Sci. Signal. 2, ra9. 10.1126/scisignal.2000016 19261596 PMC2774247

[B32] Saito H. Kubota M. Roberts R. W. Chi Q. Matsunami H. (2004). RTP family members induce functional expression of mammalian odorant receptors. Cell 119, 679–691. 10.1016/j.cell.2004.11.021 15550249

[B33] Sanz G. Schlegel C. Pernollet J. C. Briand L. (2005). Comparison of odorant specificity of two human olfactory receptors from different phylogenetic classes and evidence for antagonism. Chem. Senses 30, 69–80. 10.1093/chemse/bji002 15647465

[B34] Schiffer C. Müller A. Egeberg D. L. Alvarez L. Brenker C. Rehfeld A.. (2014). Direct action of endocrine disrupting chemicals on human sperm. EMBO Rep. 15, 758–765. 10.15252/embr.201438869 24820036 PMC4196979

[B35] Sendler E. Johnson G. D. Mao S. Goodrich R. J. Diamond M. P.. (2013). Stability, delivery and functions of human sperm RNAs at fertilization. Nucleic Acids Res. 41, 4104–4117. 10.1093/nar/gkt132 23471003 PMC3627604

[B36] Spehr J. Gelis L. Osterloh M. Oberland S. Hatt H.. (2011). G protein-coupled receptor signaling via Src kinase induces endogenous human transient receptor potential vanilloid type 6 (TRPV6) channel activation. J. Biol. Chem. 286, 13184–13192. 10.1074/jbc.M110.183525 21349844 PMC3075665

[B37] Spehr M. Gisselmann G. Poplawski A. Riffell J. A. Wetzel C. H.. (2003). Identification of a testicular odorant receptor mediating human sperm chemotaxis. Science 299, 2054–2058. 10.1126/science.1080376 12663925

[B38] Steger K. (2001). Haploid spermatids exhibit translationally repressed mRNAs. Anat. Embryol. 203, 323–334. 10.1007/s004290100176 11411307

[B39] Strünker T. Goodwin N. Brenker C. Kashikar N. D. Weyand I. Seifert R.. (2011). The CatSper channel mediates progesterone-induced Ca2+ influx in human sperm. Nature 471, 382–386. 10.1038/nature09769 21412338

[B40] Sun F. Bahat A. Gakamsky A. Girsh E. Katz N.. (2005). Human sperm chemotaxis: both the oocyte and its surrounding cumulus cells secrete sperm chemoattractants. Hum. Reprod. 20, 761–767. 10.1093/humrep/deh657 15591080

[B41] Sun Y. McGarrigle D. Huang X. (2007). When a G protein-coupled receptor does not couple to a G protein. Mol. Biosyst. 3, 849–854. 10.1039/b706343a 18000562

[B42] Thorvaldsdóttir H. Robinson J. T. Mesirov J. P. (2013). Integrative genomics viewer (IGV): high-performance genomics data visualization and exploration. Brief. Bioinformatics 14, 178–192. 10.1093/bib/bbs017 22517427 PMC3603213

[B43] Trapnell C. Williams B. A. Pertea G. Mortazavi A. Kwan G.. (2010). Transcript assembly and quantification by RNA-Seq reveals unannotated transcripts and isoform switching during cell differentiation. Nat. Biotechnol. 28, 511–515. 10.1038/nbt.1621 20436464 PMC3146043

[B44] Vanderhaeghen P. Schurmans S. Vassart G. Parmentier M. (1997a). Molecular cloning and chromosomal mapping of olfactory receptor genes expressed in the male germ line: evidence for their wide distribution in the human genome. Biochem. Biophys. Res. Commun. 237, 283–287. 10.1006/bbrc.1997.7043 9268701

[B45] Vanderhaeghen P. Schurmans S. Vassart G. Parmentier M. (1997b). Specific repertoire of olfactory receptor genes in the male germ cells of several mammalian species. Genomics 39, 239–246. 10.1006/geno.1996.4490 9119360

[B46] Vanderhaeghen P. Schurmans S. Vassart G. Parmentier M. (1993). Olfactory receptors are displayed on dog mature sperm cells. J. Cell Biol. 123(6 Pt 1), 1441–1452. 10.1083/jcb.123.6.1441 8253843 PMC2290870

[B47] Veitinger T. Riffell J. R. Veitinger S. Nascimento J. M. Triller A.. (2011). Chemosensory Ca2+ dynamics correlate with diverse behavioral phenotypes in human sperm. J. Biol. Chem. 286, 17311–17325. 10.1074/jbc.M110.211524 21454470 PMC3089573

[B48] Volz A. Ehlers A. Younger R. Forbes S. Trowsdale J.. (2003). Complex transcription and splicing of odorant receptor genes. J. Biol. Chem. 278, 19691–19701. 10.1074/jbc.M212424200 12637542

[B49] Walensky L. D. Roskams A. J. Lefkowitz R. J. Snyder S. H. Ronnett G. V. (1995). Odorant receptors and desensitization proteins colocalize in mammalian sperm. Mol. Med. 1, 130–141. 8529092 PMC2229940

[B50] Wallrabenstein I. Kuklan J. Weber L. Zborala S. Werner M.. (2013). Human trace amine-associated receptor TAAR5 can be activated by trimethylamine. PLoS ONE 8:e54950. 10.1371/journal.pone.0054950 23393561 PMC3564852

[B51] Wang E. T. Sandberg R. Luo S. Khrebtukova I. Zhang L.. (2008). Alternative isoform regulation in human tissue transcriptomes. Nature 456, 470–476. 10.1038/nature07509 18978772 PMC2593745

[B52] Wennemuth G. Westenbroek R. E. Xu T. Hille B. Babcock D. F. (2000). CaV2.2 and CaV2.3 (N- and R-type) Ca2+ channels in depolarization-evoked entry of Ca2+ into mouse sperm. J. Biol. Chem. 275, 21210–21217. 10.1074/jbc.M002068200 10791962

[B53] Wetzel C. H. Oles M. Wellerdieck C. Kuczkowiak M. Gisselmann G.. (1999). Specificity and sensitivity of a human olfactory receptor functionally expressed in human embryonic kidney 293 cells and Xenopus Laevis oocytes. J. Neurosci. 19, 7426–7433. 10460249 10.1523/JNEUROSCI.19-17-07426.1999 PMC6782526

[B54] Wolkowicz M. J. Digilio L. Klotz K. Shetty J. Flickinger C. J.. (2008). Equatorial segment protein (ESP) is a human alloantigen involved in sperm-egg binding and fusion. J. Androl. 29, 272–282. 10.2164/jandrol.106.000604 17978344 PMC2898563

[B55] Zhang X. Firestein S. (2002). The olfactory receptor gene superfamily of the mouse. Nat. Neurosci. 5, 124–133. 10.1038/nn800 11802173

[B56] Zhang X. De la Cruz O. Pinto J. M. Nicolae D. Firestein S. Gilad Y. (2007). Characterizing the expression of the human olfactory receptor gene family using a novel DNA microarray. Genome Biol. 8:r86. 10.1186/gb-2007-8-5-r86 17509148 PMC1929152

[B57] Ziegler A. Dohr G. Uchanska-Ziegler B. (2002). Possible roles for products of polymorphic MHC and linked olfactory receptor genes during selection processes in reproduction. Am. J. Reprod. Immunol. 48, 34–42. 10.1034/j.1600-0897.2002.01097.x 12322894

