# Supplementary material for: Characterization of the Olfactory Receptors Expressed in Human Spermatozoa
Source: Front Mol Biosci. 2016 Jan 7;2:73. doi: 10.3389/fmolb.2015.00073 (PMC4703994; doi:10.3389/fmolb.2015.00073)
Supplement: Supplementary file 1 [file Image1.PDF]

SUPPORTING INFORMATION

	OR	transcript orientation	detected 5' UTR
OR with FPKM >1 in at least one sperm sample	OR4N4	sense & antisense	✓
	OR52D1	antisense	×
	OR1C1	antisense	×
	OR2H1	sense & antisense	✓
	OR10J1	sense	✓
	OR8D1	antisense	×
	OR7D2	sense	✓
	OR8G5	sense	✓
	OR3A2	sense	✓
	OR7E24	sense	✓
	OR14A2	sense	×
	OR6F1	sense	✓
	OR2H2	sense	✓
	OR2C3	sense & antisense	✓
	OR52N2	sense	✓
	OR8B12	antisense	×
	OR8A1	sense & antisense	×
	OR4M1	sense & antisense	×
	OR5V1	sense	✓
	OR10A7	antisense	×
	OR1F1	sense & antisense	✓
	OR10D3	antisense	×
	OR4M2	sense & antisense	×
	OR2L2	sense	✓
	OR5M3	antisense	×
	OR8B2	sense	×
	OR6T1	sense	✓
	OR14A16	sense & antisense	✓
	OR8B3	sense	✓
	OR1K1	sense	✓
	OR10AD1	sense & antisense	✓
	OR10G6	sense	✓
	OR3A3	sense & antisense	✓
	OR6X1	antisense	×
	OR6C74	antisense	×
	OR2K2	sense & antisense	✓
	OR2T7	sense & antisense	×
	OR4N2	sense	✓
	OR2T11	antisense	×
	OR2AG2	sense & antisense	✓
	OR1F12	sense & antisense	×
	OR2A4/7	sense & antisense	×
	OR11H6	antisense	×
	OR5H6	sense & antisense	×
	OR8J1	n.a.	×

S1 Fig. Forty-five detected putative OR transcripts with an FPKM >1 in at least one sperm sample. The ORs are sorted by their mFPKM values**.**

OR	Primer sequence (5'-3')	Added restriction site
OR4N4fw	GCATATGAATTCATGAAGATAGCAAACAACACAGTAGTG	EcoRI
OR4N4rv	GCATATGCGGCCGCTCAGTTTCTTATTATAAAATCCAC	NotI
OR6B2fw	GCATATGAATTCATGAGTGGGGAGAATGTCACC	EcoRI
OR6B2rv	GCATATGCGGCCGCTCAGTGTGAAGTTTGACCCAAGC	NotI
OR2W3fw	GCAATGCGGCCGCTATGGATGGAACCAATGGCAGCACC	NotI
OR2W3rv	GCAATGGGCCCTTACTCCTTTCCTAGCTCTCTCTTCCC	ApaI
OR3A2fw	GCAATGCGGCCGCTATGGAGCCAGAAGCTGGGACC	NotI
OR3A2rv	GCAATGGGCCCTCAGGTCAGTGATCTCCTCCC	ApaI
OR2H1fw	GCATATGAATTCATGGTTAACCAAAGCTCCCCCATG	EcoRI
OR2H1rv	GCATATGCGGCCGCTTAAGCAGCTCTCCAGCTTTCCCTG	NotI
OR2H2fw	GCATATGAATTCATGGTTAACCAAAGCTCCACACC	EcoRI
OR2H2rv	GCATATGCGGCCGCTCAGCTTTGTGTGAGCCCCATTTC	NotI
OR10J1fw	GCATATGAATTCATGAAAAGAGAGAACTTTACTCTCATCA	EcoRI
OR10J1rv	GCATATGCGGCCGCTCAGGAAAACTTCCCACCAACAG	NotI

S2 Fig. Primer pairs used for cloning.

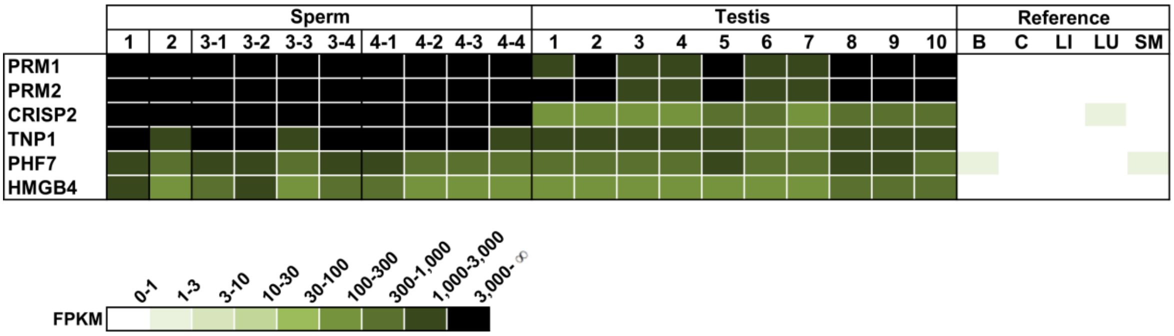

S3 Fig. Expression of housekeeping genes in human spermatozoa. The highly expressed glyceraldehyde 3-phosphate dehydrogenase (GAPDH), the moderately to highly expressed (ribosomal protein L29 (RPL29), ß-actin (ACTB) and ribosomal protein L13A (RPL13A) as well as the weakly expressed genes ß-glucuronidase (GUSB) and TATA box binding protein (TBP) are shown. The transcript levels in human spermatozoa were compared to those of ten different human testes samples as well as five reference tissues (brain (B), colon (C), liver (LI), lung (LU) and skeletal muscle (SM)).

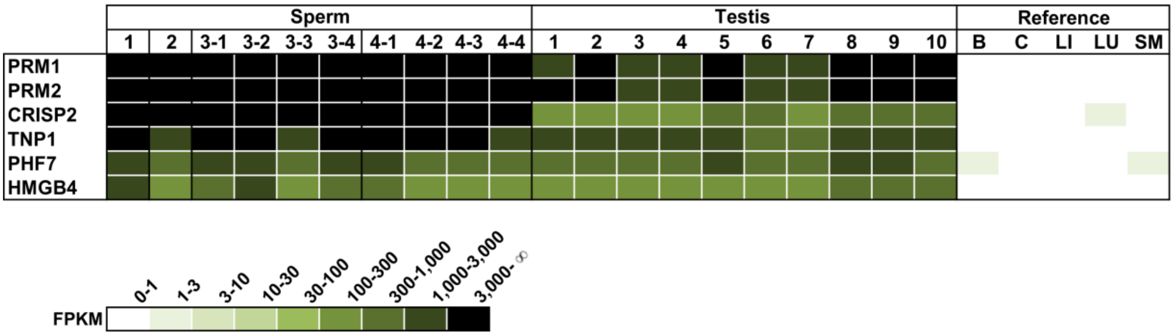

S4 Fig. Expression of sperm-associated transcripts in human spermatozoa. The typical spermatozoa-associated transcripts that were previously described are shown (30). The transcripts that are highly abundant in spermatozoa and testis and nearly absent in the reference tissues (brain (B), colon (C), liver (LI), lung (LU) and skeletal muscle (SM)) are shown, indicating the specificity of the transcriptome analysis of the sperm samples shown.

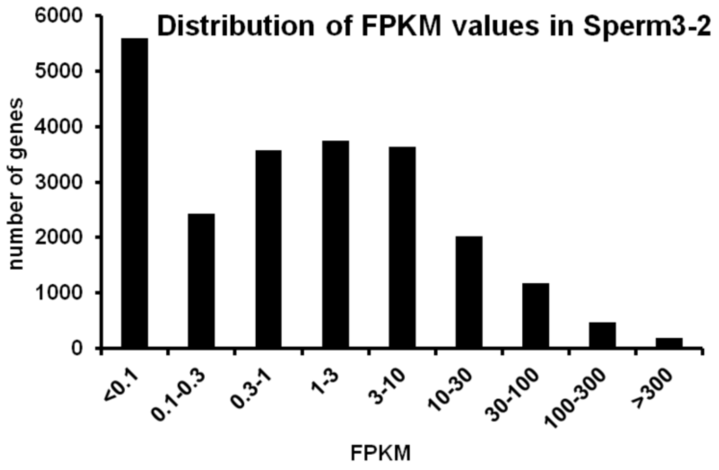

S5 Fig. Distribution of the FPKM values in human spermatozoa. To gain an estimate of the FPKM values for the expressed genes, a histogram of the FPKM distribution for the Sperm sample 3-2 was calculated. Values of 0.1-3 can be regarded as weakly expressed genes and values of 3-30 as moderately expressed genes. Values of 30-100 indicate high expression and values >100 indicate extremely high expression. Of the ~23,000 analyzed genes, expression at >0.1 FPKM was detected for ~17,000 genes; the mRNAs for ~600 of these genes were extremely highly expressed with FPKM values >100.

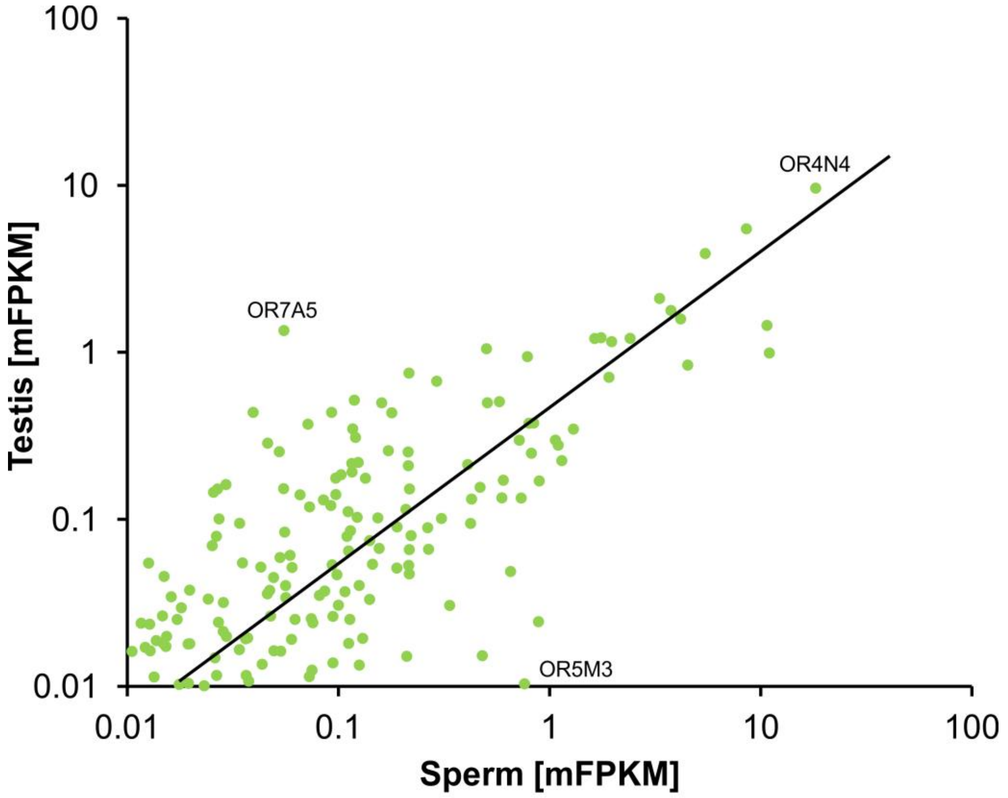

S6 Fig. Comparison of the olfactory transcriptome from sperm and testis. The expression pattern correlations for all putative OR transcripts in sperm and testis samples are plotted. The mFPKM values with no distinction between antisense and sense are shown. OR4N4 showed a strong correlation. OR7A5 is more highly expressed in the testis than in sperm, whereas OR5M3 is more highly expressed in sperm than in testis. Pearson's correlation coefficient = 0.86.

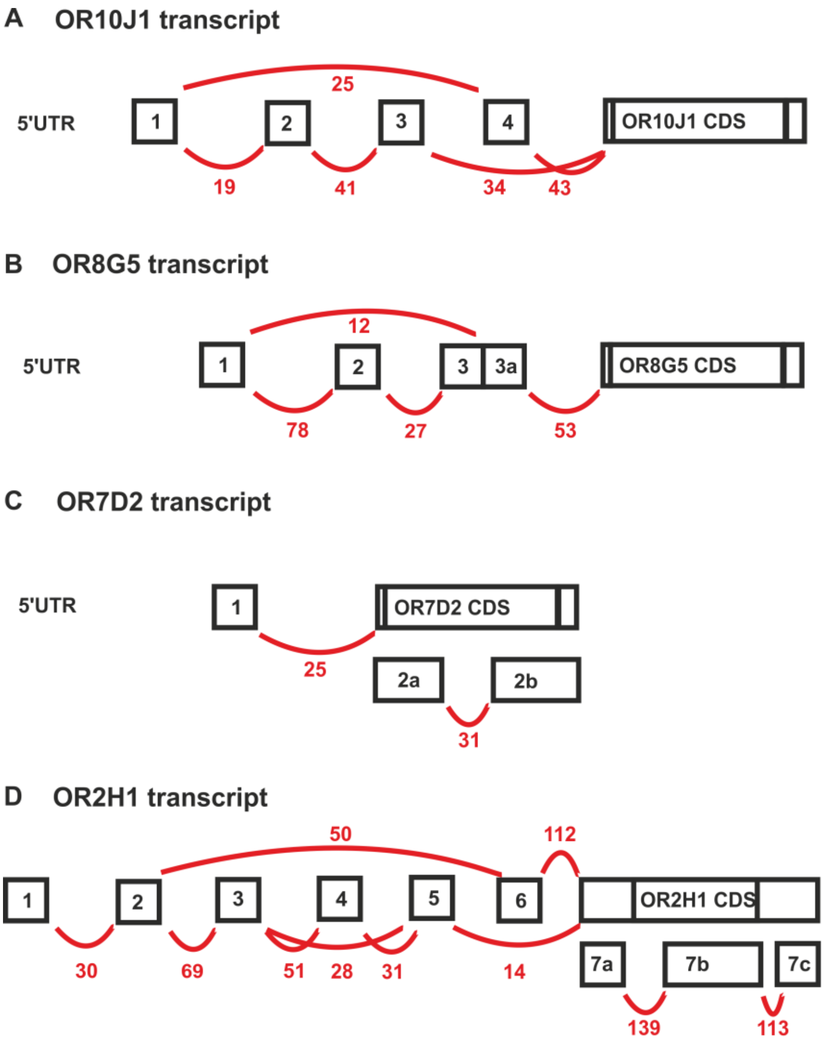

OR10J1	5` end	3` end	length (bp)
Exon 1	159367299	159367320	22
Exon 2	159375095	159375186	92
Exon 3	159376037	159376203	167
Exon 4	159401074	159401261	188
Exon 5	159409566	159410731	1166
OR10J1 CDS	159409582	159410511	930

OR8G5	5` end	3` end	length (bp)
Exon 1	124111892	124112104	213
Exon 2	124118552	124118631	80
Exon 3	124126168	124126525	358
Exon 3a	124126399	124126525	127
Exon 4	124134814	124136010	1197
OR8G5 CDS	124134828	124135736	909

OR7D2	5` end	3` end	length (bp)
Exon 1	9294668	9294686	19
Exon 2	9296445	9297442	998
Exon 2a	9296445	9296757	313
Exon 2b	9297221	9297442	222
OR7D2 CDS	9296458	9297396	939

OR2H1	5` end	3` end	length (bp)
Exon 1	29424960	29424984	25
Exon 2	29426231	29426346	116
Exon 3	29427052	29427146	95
Exon4	29427249	29427363	115
Exon 5	29427798	29427960	163
Exon6	29428181	29428231	51
Exon 7	29429274	29432038	2765
Exon 7a	29429274	29429527	254
Exon7b	29429781	29430510	730
Exon7c	29431526	29432038	513
OR2H1 CDS	29429547	29430497	951

S7 Fig. Analysis of the OR transcripts detected in human spermatozoa. Schematic representation of the newly identified 5’UTRs. The detected exons are indicated by black boxes (exon). The coding exon is indicated by CDS (Coding Sequence) and the splice junctions as red arcs. We showed the 5’UTRs detected for OR10J1 (A), OR8G5 (B), OR7D2 (C), and OR2H1 (D). The red numbers indicate the reads supporting the splice junctions.

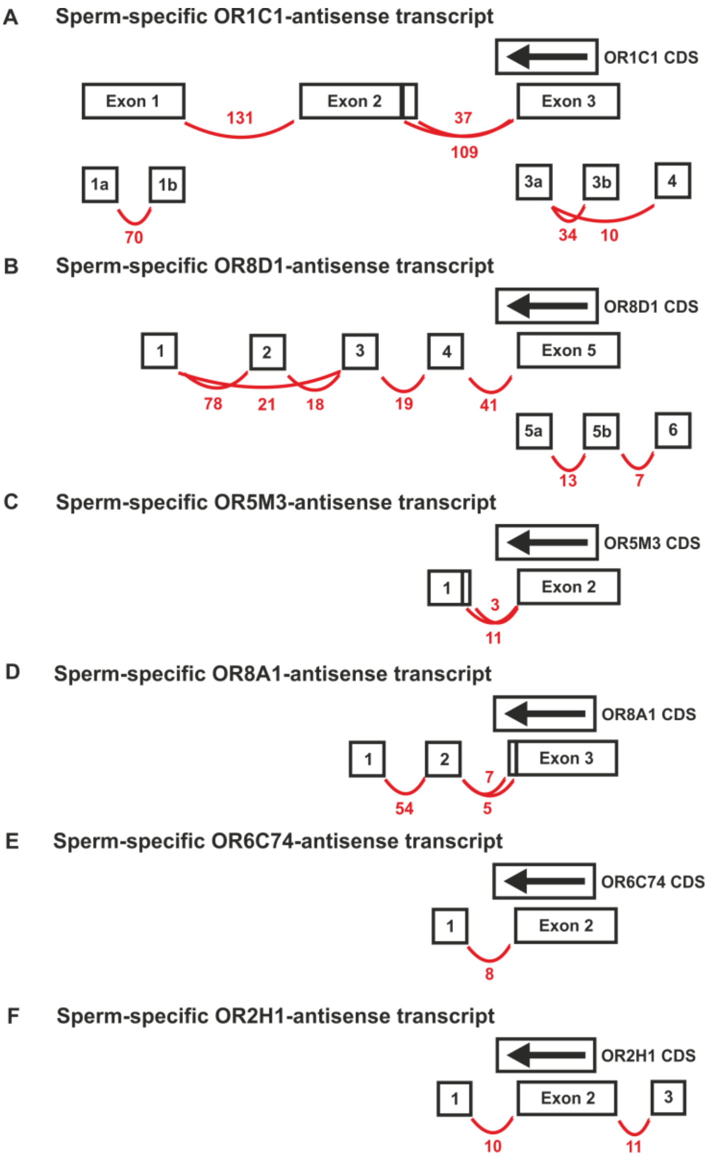

OR1C1-as	5` end	3` end	length (bp)
Exon 1	247920836	247921754	919
Exon 1a	247920836	247921250	415
Exon 1b	247921376	247921754	379
Exon 2	247909505	247909814	310
Exon 2a	247909505	247910044	540
Exon 3	247803030	247803731	702
Exon 3a	247803030	247803249	220
Exon 3b	247803352	247803731	380
Exon 4	247932547	247932658	112
OR1C1 CDS	247920764	247921708	945

OR8D1-as	5` end	3` end	length (bp)
Exon 1	124111892	124112104	213
Exon 2	124118552	124118631	80
Exon 3	124158989	124159075	87
Exon 4	124176659	124176781	123
Exon 5	124179790	124181572	1783
Exon 5a	124179790	124180030	241
Exon 5b	124180407	124181572	1166
Exon 6	124183617	124183655	39
OR8D1 CDS	124180662	124179736	927

OR5M3-as	5` end	3` end	length (bp)
Exon 1	56236253	56236463	211
Exon 1a	56236253	56236422	170
Exon 2	56237107	56237998	892
OR5M3 CDS	56237050	56237973	924

OR8A1-as	5` end	3` end	length (bp)
Exon 1	124448185	124447945	241
Exon 2	124445667	124445577	91
Exon 3	124440931	124439982	950
Exon 3a	124440852	124439982	871
OR8A1 CDS	124439965	124440945	981

OR6C74-as	5` end	3` end	length (bp)
Exon 1	55641938	55640783	1156
Exon 2	55652243	55652055	189
OR6C74 CDS	55641072	55642010	939

OR2H1-as	5` end	3` end	length (bp)
Exon 1	29432040	29431524	517
Exon 2	29430510	29429302	1209
Exon 3	29429008	29428834	175
OR2H1 CDS	29429547	29430497	951

G Location of the antisense exon relative to the coded amino acid in the OR

```
     ......... TM6 ...........      ....... TM7 ........

OR1C1
<<<<<<<<<<<< exon of the antisense transcript <<<<<<<<<<<<-5′
RAVSTCSCHLSVVVLFYGTAIAVYFSPSSPHMPESDTLSTIMYSMVAPMLNPFIYTLRNRDMKRGLQKMLLKCTVFQQQ

OR5M3
<<<<<<<<<<<< exon of the antisense transcript <<<<<<<<<<<<-5′
KAFSTCGSHLTAVIIFYGTLIFMYLRRPTEESVEQGKMVAVFYTTVIPMLNPMIYSLRNKDVKKAMMKVISRSC

OR8D1
<<<<<<<<<<<< exon of the antisense transcript <<<<<<<<<<<<-5′
KAFGTCSSHLMAVVIFFGSITFMYFKPPSSNSLDQEKVSSVFYTTVIPMLNPLIYSLRNKDVKKALRKVLVGK

OR6C74
<<<<<<<<<<<< exon of the antisense transcript <<<<<<<<<<<<-5′
KAFSTCSSHMVVVSISYGSCIFMYVKPSAKERVSLNKGIALLSTSVAPMLNPFIYTLRNKQVKDVFKHTVKKIELFSMK

OR2H1
<<<<<<<<<<<< exon of the antisense transcript <<<<<<<<<<<<-5′
KAFGTCSSHLTVVTLFYSSVIAVYLQPKNPYAQGRGKFFGLFYAVGTPSLNPLVYTLRNKEIKRALRRLLGKERDSRESWRAA

OR5H6
<<<<<<<<<<<< exon of the antisense transcript <<<<<<<<<<<<-5′
KAVSTCGAHLLSVSLYYGPLTFKYLGSASPQADDQDMMESLFYTVIVPLLNPMIYSLRNKQVIASFTKMFKSNV

OR8A1
<<<< exon of the antisense transcript <<<<<<<-5′
KAFSTCSSHLAAVGMFYGSTAFMYLKPSTISSLTQENVASVFYTTVIPMLNPLIYSLRNKEVKAAVQKTLRGKLF
```

S8 Fig. Identification of sperm-specific OR-antisense transcripts. A detailed analysis with the IGV for the mRNA-Seq and stranded RNA-Seq data revealed the expression of unannotated antisense transcripts relative to the respective OR transcript. The detected exons are indicated by black bars (exon). The coding exon is indicated by CDS (Coding Sequence)

and the splice junctions as red arcs. The red numbers indicate the reads supporting the splice junctions. Sperm-specific antisense transcripts are shown. For OR1C1 (A), OR8D1 (B), OR5M3 (C), OR8A1 (D), OR6C74 (E), and OR2H1 (F). The chromosome coordinates are shown for all newly indentified transcripts. (G) Location of the antisense exon relative to the coded amino acid in the OR.

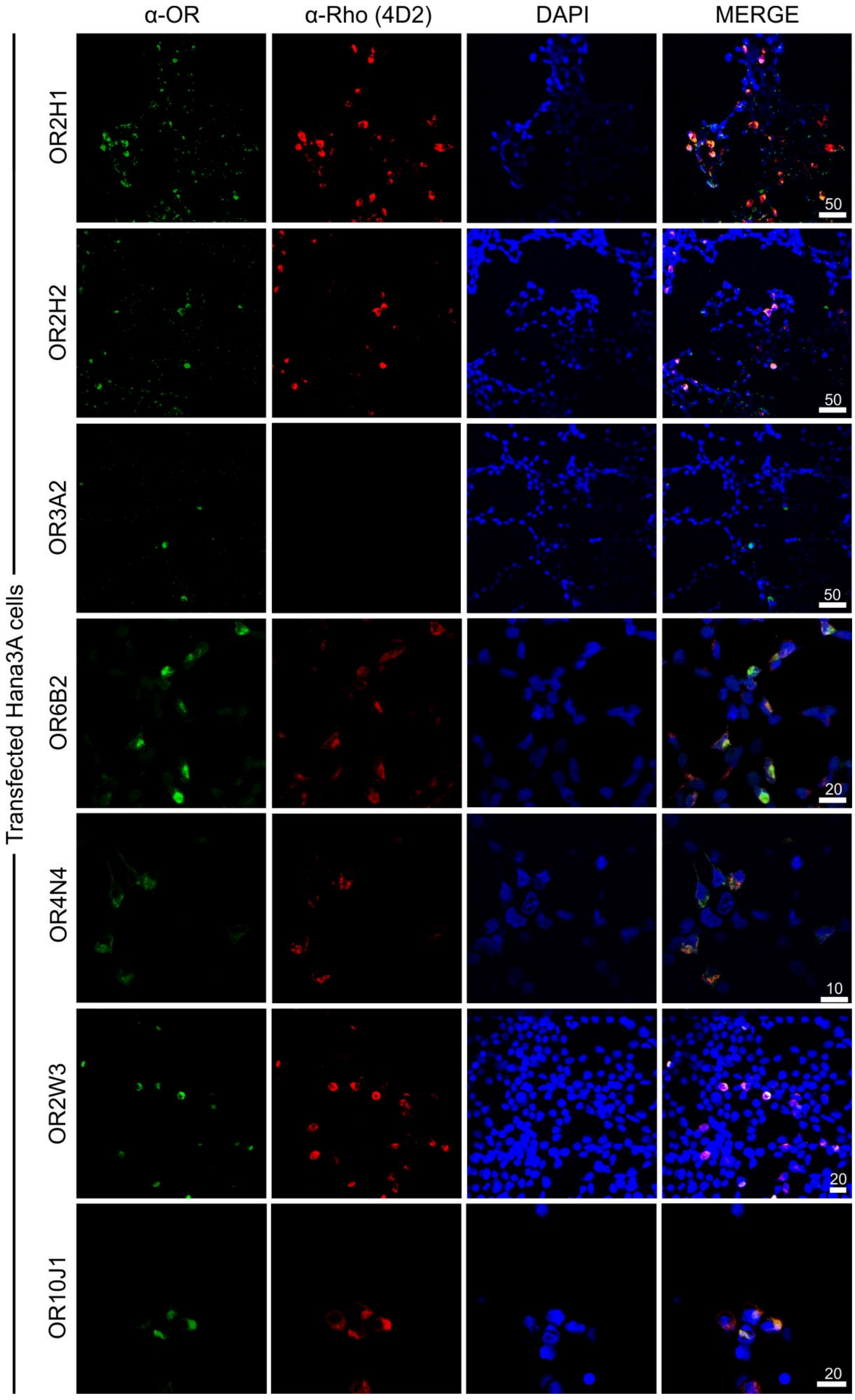

α-OR
α-Rho (4D2)
DAPI
MERGE
Transfected Hana3A cells
OR2H1
OR2H2
OR3A2
OR6B2
OR4N4
OR2W3
OR10J1
50
50
50
20
10
20
20

S9 Fig. The α-OR antibodies specifically detect recombinantly expressed ORs in Hana3A cells. Immunostaining of Hana3A cells transiently transfected with the respective OR. The cells were stained with a specific α-OR antibody (green) and a rhodopsin-antibody (rho, red). For OR3A2 no rho-tagged OR plasmid was available. The specificity of the α-OR51E1 and α-OR51E2 antibodies was proven elsewhere (data not shown). DAPI staining (blue) was used to confirm the number and location of the cell nuclei. The numbers over the scale bars are in µm.

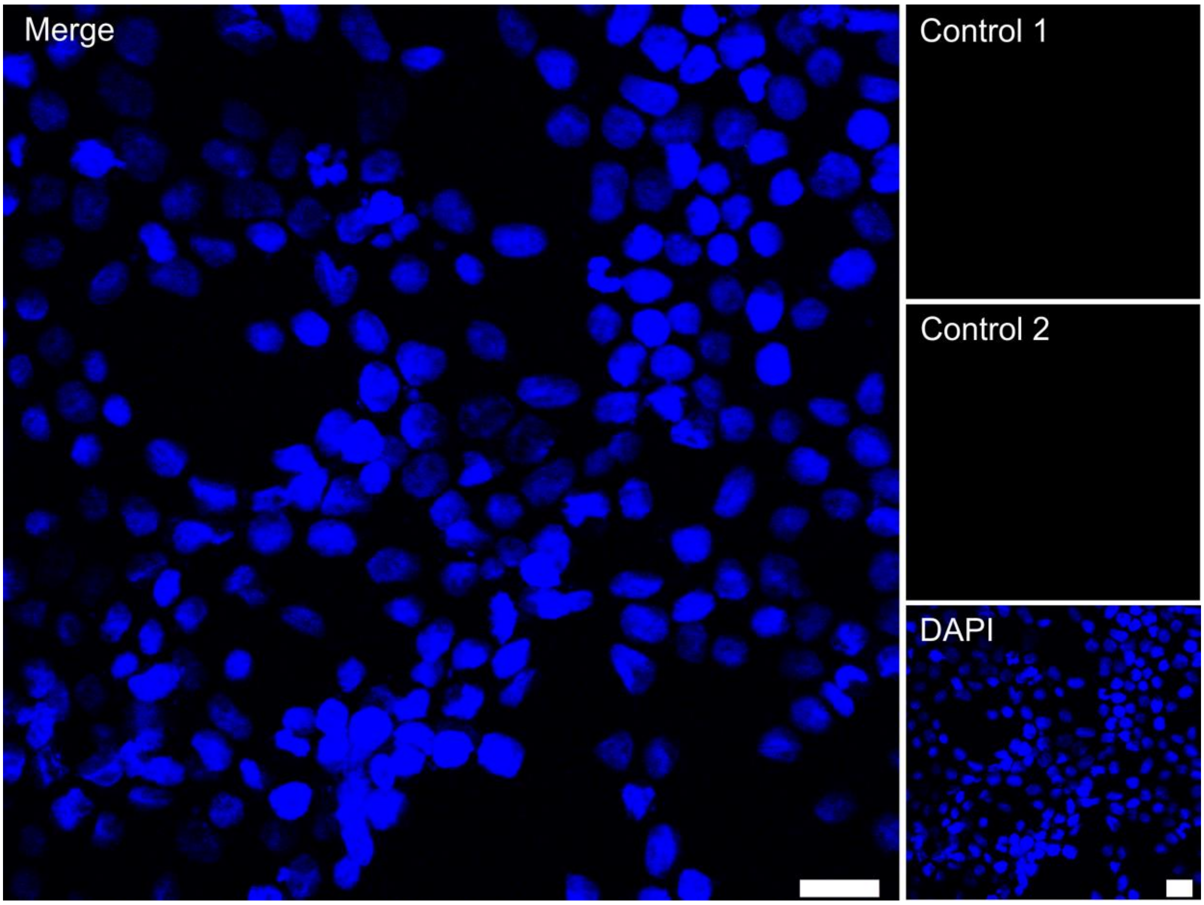

S10 Fig. Secondary antibody control in Hana3A cells. Control staining was performed without a primary antibody. The secondary antibodies Alexa Fluor 488 Goat Anti-Rabbit (Control 1) and Alexa Fluor 546 Goat Anti-Mouse (Control 2) alone did not produce any specific staining. Scale bar: 20 µm.

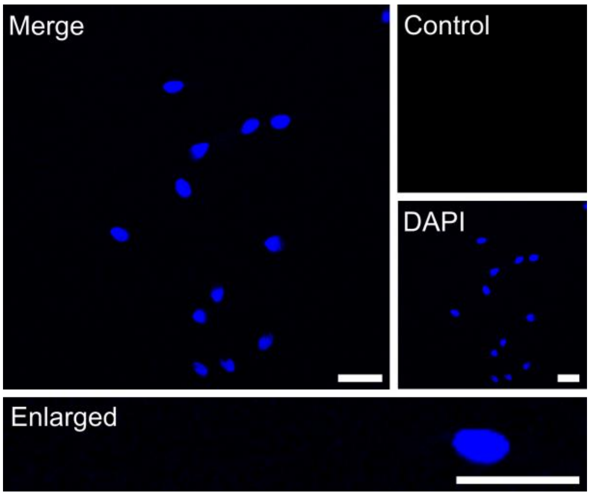

S11 Fig. Secondary antibody control in human spermatozoa. Control staining was performed without a primary antibody. The secondary antibody (Alexa Fluor 488 Goat Anti-Rabbit) alone did not produce any specific staining (Control). Scale bar: 10 µm.

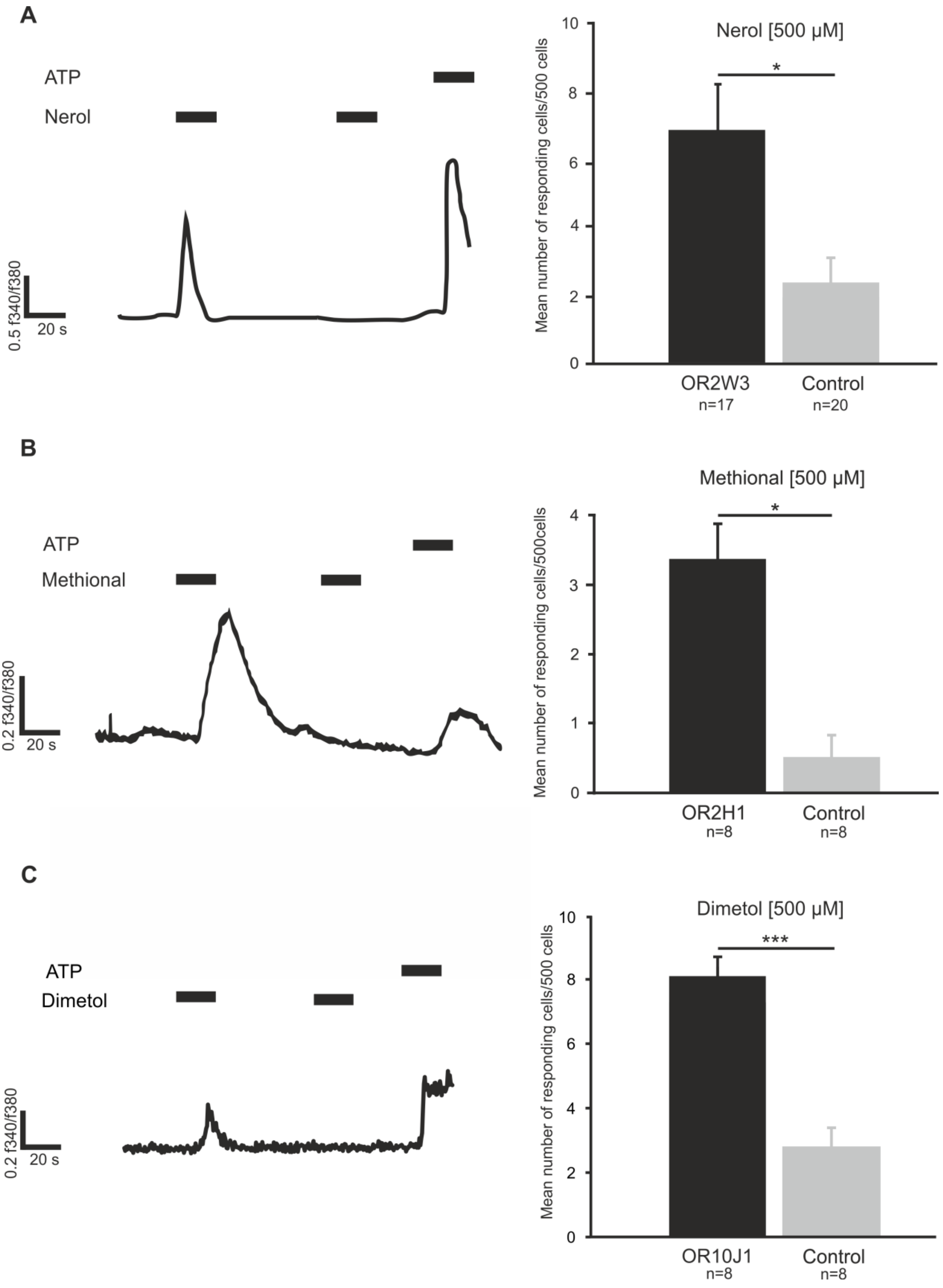

S12 Fig. OR2W3, OR2H1 and OR10J1 can be activated by specific odorants. *Left:* Representative calcium imaging traces of Hana3A cells transiently transfected with (A) OR2W3, (B) OR2H1, or (C) OR10J1 that were stimulated for 48 h after transfection with the respective odorant (500 µM). The black bars indicate the stimulus duration. ATP-induced activation (100 µM) of cells controls for cell viability. *Right:* Quantification of the

deorphanization experiments of OR-expressing Hana3A cells with (A) nerol, (B) methional, or (C) dimetol. The mean numbers of the cells (black) responding to the respective odorant (500 µM) were compared to the mean number of the mock-transfected cells responding to the stimulus (control, grey) for each measurement. n: number of measurements. Approximately 500 cells per measurement were investigated. The data are shown as the means ± SEM. Chi-squared test: *p<0.05; **p<0.01; ***p<0.001.

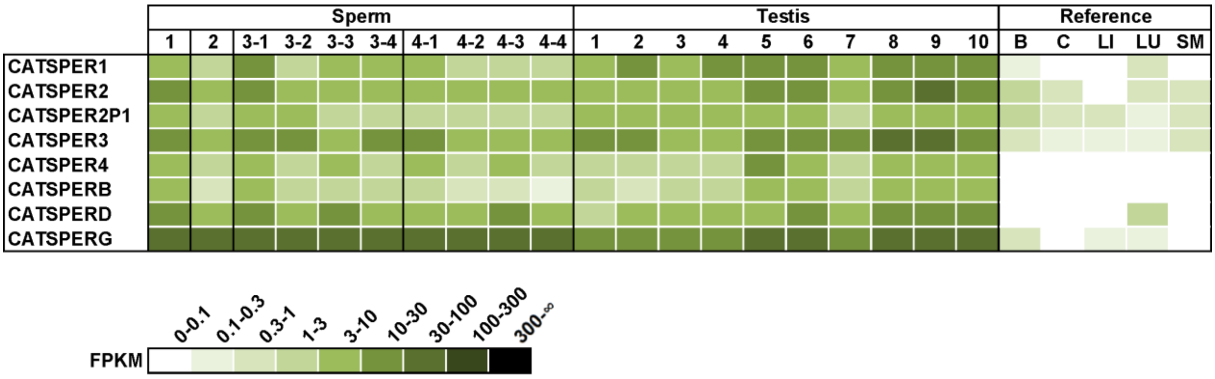

S13 Fig. Expression of CatSper in human spermatozoa. The expression profile of sperm-associated cation channels CATSPER subunits as well as the auxiliary CATSPERD, also known as TMEM146.

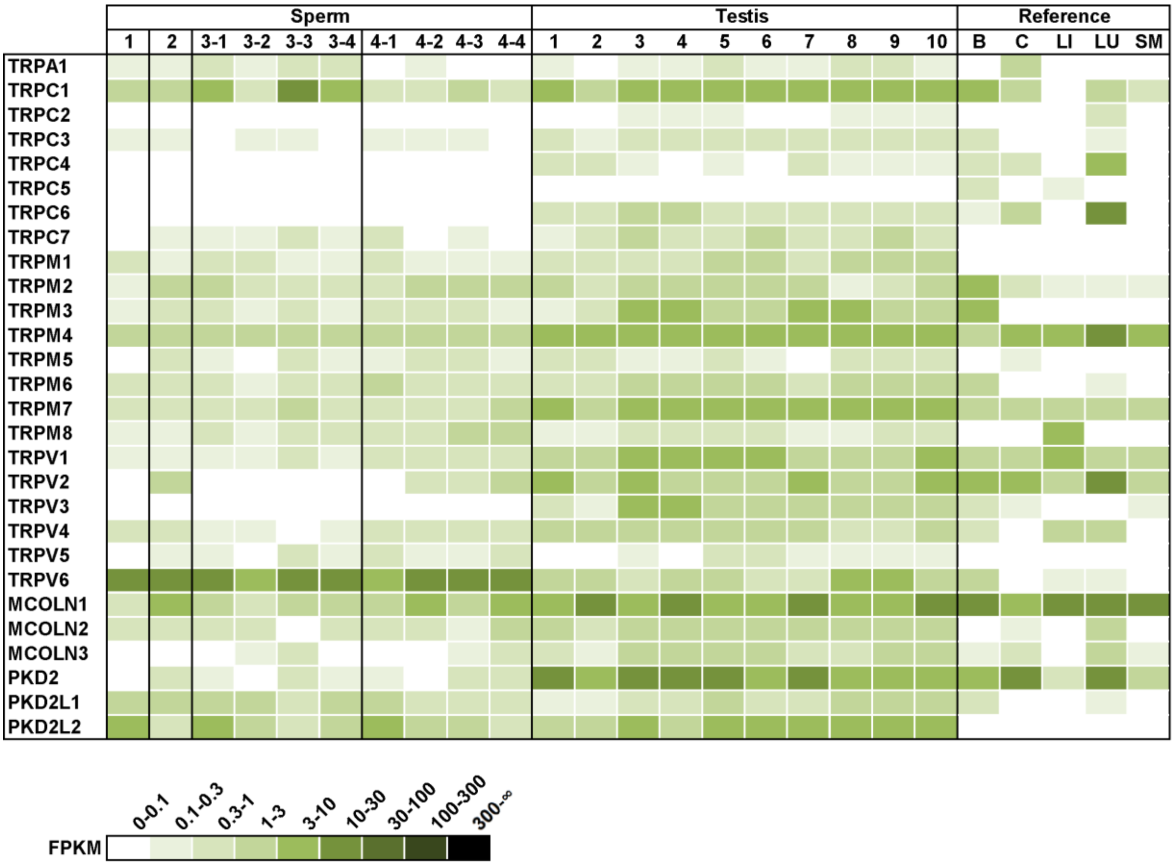

S14 Fig. Expression of TRP in human spermatozoa. The FPKM values for TRP channel transcripts in human spermatozoa are shown.

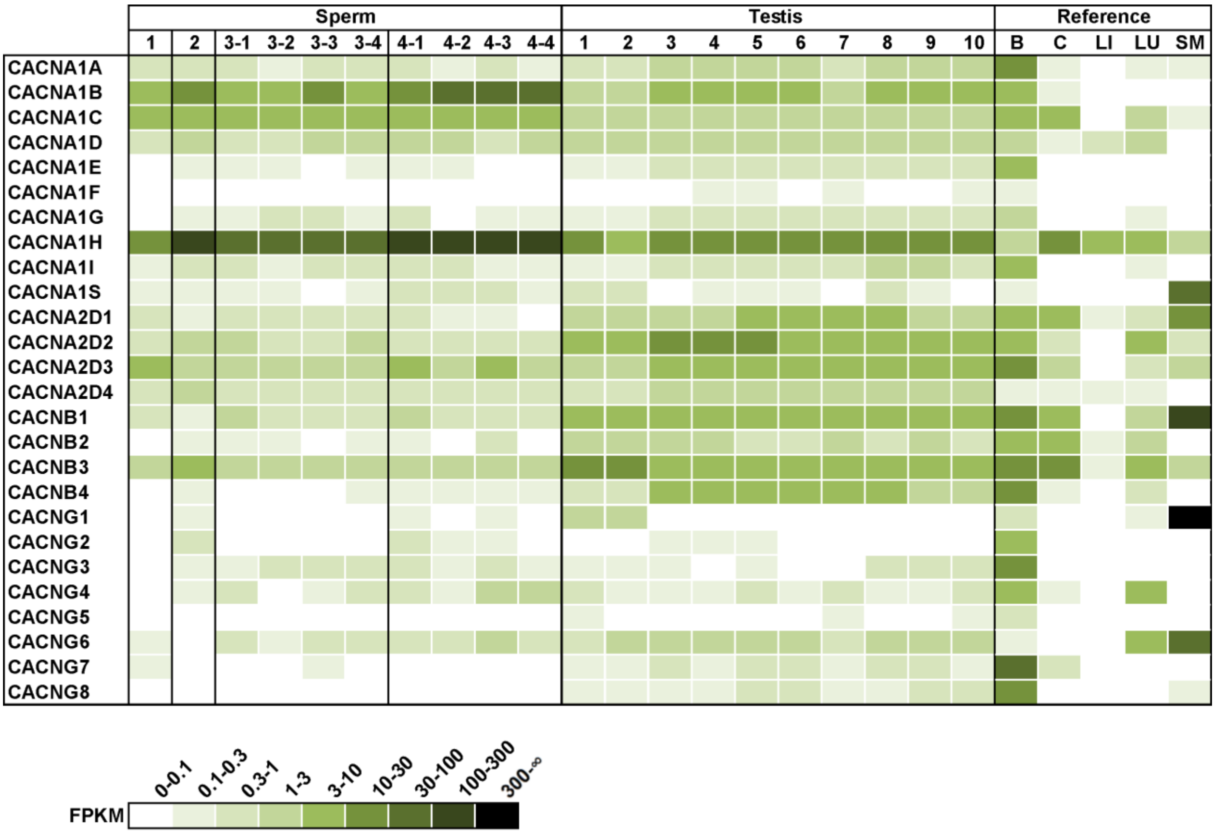

S15 Fig. Expression of voltage-dependent calcium channel subunits in human spermatozoa.

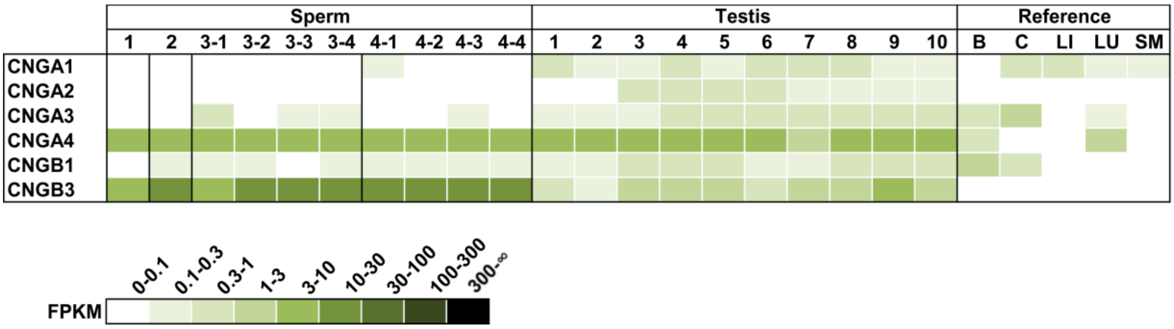

S16 Fig. Expression of CNG channel subunits in human spermatozoa.

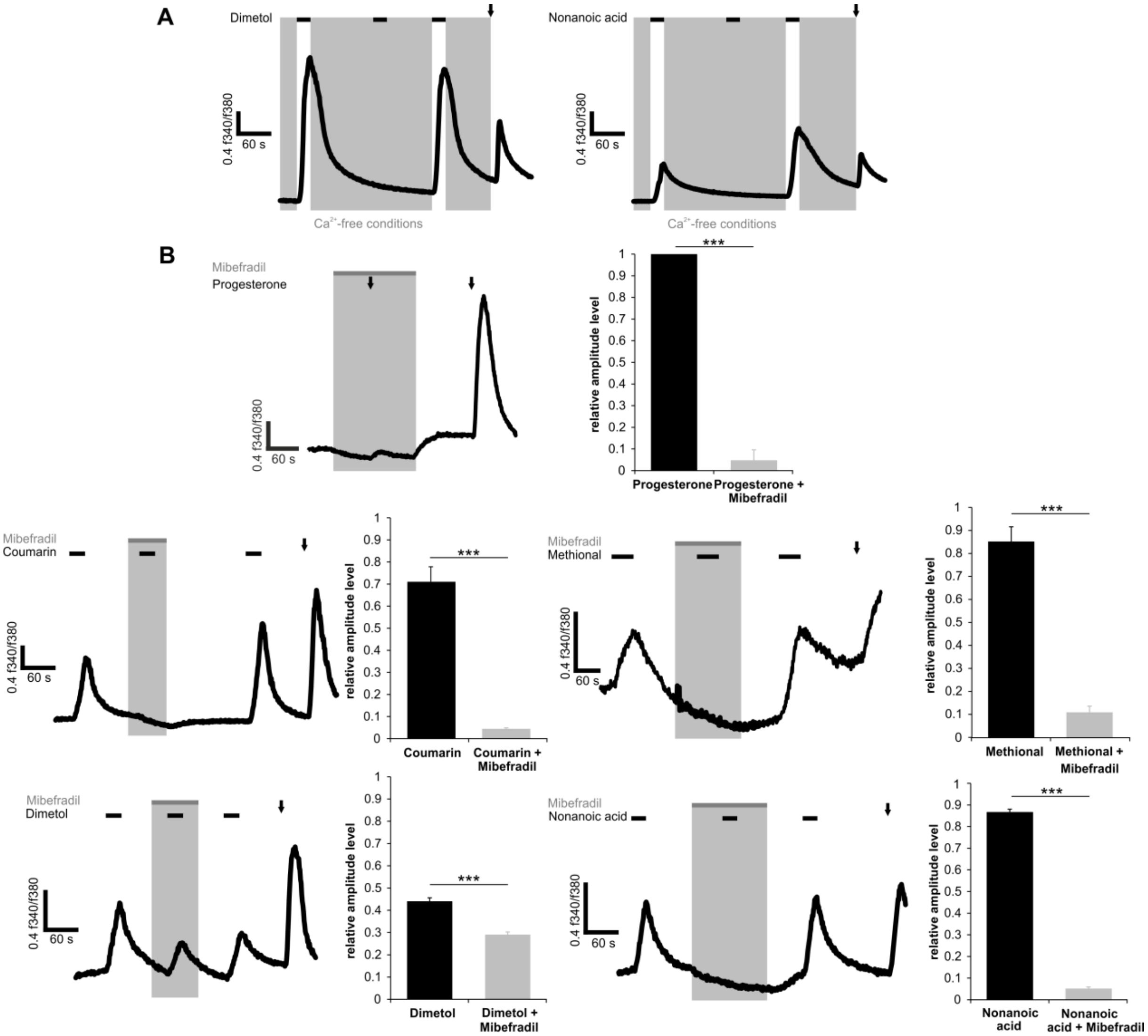

S17 Fig. Odorant-induced Ca^{2+} signals depend on extracellular calcium and can be inhibited by a calcium channel blocker. Exemplarily calcium imaging traces of human spermatozoa are shown. Progesterone (10 µM) was used as positive control at the end of each measurement (arrow). (A) Measurements with a Ca^{2+}-free extracellular solution (10 mM EGTA, marked by grey bars). Dimetol: 100 µM (n=43); Nonanoic acid: 300 µM (n=29). (B**)** Odorant-induced Ca^{2+} signals were inhibited by mibefradil (30 µM). Inhibitor measurements with progesterone (10 µM) were used as a positive control (n=83). Progesterone (10 µM was used as a positive control (arrow) at the end of each odorant-measurement. The grey bars indicate the application of the inhibitor application. The black bars indicate the duration of the odorant application. Quantification of odorant-induced Ca^{2+} signals from human spermatozoa in the inhibitor measurements relative to control are shown on the right. Coumarin: 100 µM (n=110); Dimetol 300 µM (n=182); Methional: 300 µM (n=25); Nonanoic acid: 300 µM (n=240); n= number of cells. The data are shown as the means ± SEM. Wilcoxon-Mann-Whitney-Test: *p<0.05; **p<0.01; ***p<0.001.
